# The Influence of Ionizing Radiation on Quantification for In Situ and Operando Liquid‐Phase Electron Microscopy

**DOI:** 10.1002/adma.202415728

**Published:** 2025-02-21

**Authors:** Birk Fritsch, Serin Lee, Andreas Körner, Nicholas M. Schneider, Frances M. Ross, Andreas Hutzler

**Affiliations:** ^1^ Helmholtz Institute Erlangen‐Nürnberg for Renewable Energy (IET‐2) Forschungszentrum Jülich GmbH Cauerstr. 1 91058 Erlangen Germany; ^2^ Department of Materials Science and Engineering Massachusetts Institute of Technology 77 Massachusetts Avenue Cambridge MA 02139 USA; ^3^ Department of Chemical and Biological Engineering Friedrich‐Alexander‐Universität Erlangen‐Nürnberg Immerwahrstraße 2a 91054 Erlangen Germany; ^4^ Renata Global Cambridge MA 02138 USA

**Keywords:** electron beam effects, in situ methods, kinetic modeling, liquid cell or liquid‐phase transmission electron microscopy (LC‐TEM or LP‐TEM), radiation chemistry

## Abstract

The ionizing radiation harnessed in electron microscopes or synchrotrons enables unique insights into nanoscale dynamics. In liquid‐phase transmission electron microscopy (LP‐TEM), irradiating a liquid sample with electrons offers access to real space information at an unmatched combination of temporal and spatial resolution. However, employing ionizing radiation for imaging can alter the Gibbs free energy landscape during the experiment. This is mainly due to radiolysis and the corresponding shift in chemical potential; however, experiments can also be affected by irradiation‐induced charging and heating. In this review, the state of the art in describing beam effects is summarized, theoretical and experimental assessment guidelines are provided, and strategies to obtain quantitative information under such conditions are discussed. While this review showcases these effects on LP‐TEM, the concepts that are discussed here can also be applied to other types of ionizing radiation used to probe liquid samples, such as synchrotron X‐rays.

## Graphical abstract

Liquid‐phase transmission electron microscopy enables visualization of nanoscale processes involving liquid media. Yet, it suffers from beam effects, such as radiolysis of the liquid, sample heating, and membrane charging. This review summarizes beam effect fundamentals, describes modeling and assessment, and illustrates handling strategies. The findings are transferable to other ionizing radiation techniques using, for example, γ‐ or X‐rays.

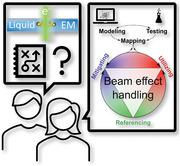

## 1 Introduction

The decades‐old concept of enclosing a liquid specimen in a small cavity to perform transmission electron microscopy (TEM)^[^ ^1^ ^]^ was implemented in a practical way in 2003 by exploiting silicon microfabrication technology to develop the so‐called liquid cell (LC).^[^ ^2^ ^]^ This enabled dynamic processes of nanomaterials in liquids to be recorded, including under electrochemical stimulus, with the exciting combination of resolution in space and time offered by TEM. The technique, referred to as liquid‐cell or liquid‐phase transmission electron microscopy (LP‐TEM), became a game changer for real‐time observations of processes in liquids down to the atomic scale, as was impressively shown soon afterward by experiments imaging nanoparticle nucleation and growth^[^ ^3^ ^]^ and oriented attachment of nanostructures at atomic resolution.^[^ ^4^ ^]^ The subsequent demonstration that graphene is also suitable for enclosing liquid specimens in TEM^[^ ^5^ ^]^ has facilitated numerous other studies, including at single adatom resolution in aqueous systems.^[^ ^6^ ^]^

LP‐TEM is seen as a method that can offer unique insights into nanoscale dynamics with opportunities in fields spanning from biology^[^ ^7^, ^8^, ^9^, ^10^ ^]^ and soft matter^[^ ^9^, ^11^, ^12^ ^]^ to nanoparticle evolution,^[^ ^13^, ^14^, ^15^ ^]^ and applications including catalysis,^[^ ^16^, ^17^, ^18^, ^19^, ^20^, ^21^, ^22^, ^23^ ^]^ battery research,^[^ ^24^, ^25^, ^26^, ^27^, ^28^ ^]^ and corrosion science.^[^ ^29^, ^30^ ^]^ However, an unavoidable aspect of the use of LP‐TEM is the fact that the irradiation required for imaging induces effects on the experiment.^[^ ^16^, ^17^, ^18^, ^19^, ^20^, ^31^, ^32^ ^]^

Recent reviews unanimously name irradiation effects as one of three obstacles that must be overcome if we are to apply LP‐TEM as a quantitative standard characterization tool.^[^ ^16^, ^20^, ^22^, ^29^, ^33^ ^]^ Our insufficient understanding of electron irradiation effects on the solution chemistry constitutes perhaps the most critical and universal obstacle; others are the limited temporal and spatial resolution using liquid holder systems, which is mainly caused by the LC architecture and sample dose sensitivity, and the need for a strategy for obtaining quantitative chemical information in real‐time. We emphasize that beam‐induced alterations are not exclusive to LP‐TEM but apply to any in situ or *operando* technique that harnesses ionizing radiation, such as X‐rays or neutrons, to study dynamics in liquid.^[^ ^34^, ^35^, ^36^, ^37^ ^]^ However, the assessment of radiation artifacts and, thus, their appropriate handling are not discussed synergistically within these heterogeneous communities.^[^ ^35^, ^37^ ^]^

In this review, we provide insights into radiation damage in liquids by critically discussing recent achievements that aim to understand, mitigate, and exploit electron beam‐driven effects in LP‐TEM. Energy transfer always accompanies electron microscopy, but the specific cases that arise during LP‐TEM experiments require dedicated handling, particularly regarding radiolysis. Hence, we provide guidelines for experimental design to facilitate quantitative outcomes from LP‐TEM. As electron radiation damage in general, and electron radiation chemistry in particular, are similar in concept to phenomena associated with other radiation sources, most of the discussion in this review is transferable to other ionizing radiation methods used for nanoscale *operando* or in situ studies.

We start with an introduction to the fundamentals of inelastic scattering, followed by a discussion of radiation chemistry and interpretation. We then review approaches to access the outcomes of radiation chemistry, both experimentally and via kinetic modeling, during LP‐TEM. By providing guidelines on how to perform radiation chemistry simulations successfully and highlighting best practices in experimental design to account for beam effects, we sketch a framework for how to handle beam‐induced effects constructively. We aim for this to facilitate LP‐TEM experimentation that leads to realistic and quantitative information. We conclude by discussing open questions and further challenges.

As the early stage of beam‐induced artifacts in LP‐TEM has been reviewed elsewhere,^[^ ^15^, ^38^, ^39^ ^]^ we focus on recent developments alongside benchmark literature. Furthermore, we intentionally do not discuss how to set up an LP‐TEM experiment from a technical viewpoint,^[^ ^40^, ^41^, ^42^ ^]^ the physical limits of image resolution,^[^ ^43^ ^]^ or recent advances in LC architectures, nor provide an in‐depth discussion of LP‐TEM applications^[^ ^44^ ^]^ or data processing strategies^[^ ^13^, ^45^ ^]^ as many of these aspects have been elucidated elsewhere, including in peer‐reviewed video tutorials.^[^ ^46^, ^47^, ^48^, ^49^, ^50^, ^51^, ^52^ ^]^

## 2 From Irradiation to Absorbed Power

When we employ electrons to study aqueous dynamics, we encounter phenomena caused by the probe itself through inelastic scattering. To quantify inelastic effects during irradiation, we introduce fundamentals of energy transfer from radiation to matter and provide guidelines to translate experimentally accessible parameters to the absorbed energy. Energy transfer from radiation to matter occurs randomly along the trajectory (track) by inelastic scattering processes. The average distance between these scattering events, referred to as the inelastic mean free path *λ*_IMFP_, is defined by the inelastic scattering cross‐section *σ*_INEL_ and the quantity of molecules per volume, defined by the molecular weight *M* and density *ρ* of the medium (*N_A_* is Avogadro's constant):^[^ ^53^ ^]^

(1) $$\lambda_{\mathrm{IMFP}}=\frac{M}{N_{A}\rho \sigma_{\mathrm{INEL}}}\;\;$$

For electrons, photons, and fast charged particles, the energy transfer per scattering event is small and can be approximated linearly along the trajectory. The magnitude of energy uptake of a medium due to irradiation is quantified by the linear energy transfer (LET) value. Equivalently, the loss of energy *E* experienced by the radiation along the trajectory *s* is quantified by the stopping power *S*,^[^ ^54^ ^]^ herein normalized to *ρ*:^[^ ^55^ ^]^

(2) $$S=\frac{dE}{ds}\;\frac{1}{\rho }\;\;$$

It is important to keep in mind that LET values and *S* are specific to the nature of the material and the irradiation type. The LET values of fast electrons, hard X‐rays, and γ‐photons are similar. These values define the nature of the physical stage of water radiolysis, which we discuss in Section 3.1. Therefore, these so‐called low LET radiation types cause similar radiation chemistry, which is contrasted by high LET irradiation such as α‐particles or protons.^[^ ^54^, ^56^ ^]^ Nonetheless, scattering cross sections and inelastic mean free paths can differ substantially.

The total energy absorbed by matter is called dose and is typically given in units of $\left[\frac{J}{\mathrm{kg}}\right]$. The temporal change of the dose is called dose rate (*ψ*), which is a critical factor for radiation chemistry assessment. The dose rate is expressed in units of Gray per second $\left(\left[1\;\frac{\mathrm{Gy}}{s}=\frac{J}{\mathrm{kg}\cdot s}=\frac{W}{\mathrm{kg}}\;\right]\right)$. The existence of a steady state in radiation chemistry renders *ψ* a crucial parameter, as discussed below.

Naturally, dose and dose rate physically differ from radiation intensity or radiant flux. Many tools and methods exist for calculating the dose during photon‐based methods, such as synchrotron radiation,^[^ ^57^, ^58^ ^]^ X‐ray exposure,^[^ ^59^, ^60^ ^]^ or medical applications^[^ ^61^, ^62^ ^]^ to which we refer the interested reader. However, for electron microscopy we emphasize that a dose rate is not equivalent to the electron‐flux density *ϕ*, often provided by the microscope user interface in units of $\frac{e^{-}}{nm^{2}s}$ or $\frac{e^{-}}{{Å}^{2}s}$. *ϕ* is an experimentally controllable quantity that can be converted to *ψ*. Assuming that the track of the primary electron equals the liquid layer thickness *z_l_*, the following equation holds:^[^ ^55^, ^63^ ^]^

(3) $$\psi =\frac{\phi }{e}\;S\left(1+\frac{z_{l}}{\lambda_{\mathrm{IMFP}}}\right)\;\;$$

**Figure 1:**
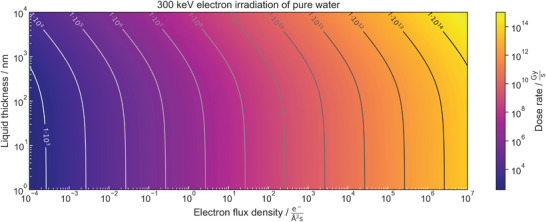
The dose rate experienced by pure water when exposed to 300 keV electrons is displayed as a function of the electron‐flux density and the thickness of the liquid layer. The level line labels denote dose rates in $\frac{\mathrm{Gy}}{s}$. Calculations are performed using Equation (3) after ref. [63].

To convert the electron‐flux density to a number density, *ϕ* is divided by the elementary charge *e*. All these parameters depend on the chosen experimental conditions, including the kinetic energy of the electrons in the beam. In the case of pure water or highly‐diluted aqueous systems irradiated with an acceleration voltage of 300 kV ($S=2.36\;\frac{\mathrm{MeV}{\left(\mathrm{cm}\right)}^{2}}{g}$,^[^ ^64^ ^]^ *λ*_IMFP_ =  380 nm),^[^ ^65^ ^]^ this relationship is visualized in **Figure** **1**.

We would like to draw the reader's attention to several key aspects of the discussion above. First, the dose rate is generally high in practical microscopy and imaging always comes along with dose deposition: in cryo‐TEM, inelastic scattering occurs about three times more frequently than elastic scattering when the acceleration voltage is between 100 and 300 kV.^[^ ^34^, ^66^, ^67^ ^]^ As the inelastic mean free path of electrons in water appears to be comparable to that in amorphous ice,^[^ ^65^, ^68^ ^]^ we assume this to hold for LP‐TEM as well. Consequently, even “low” dose rates during LP‐TEM still deposit substantial power to the sample that can cause beam artifacts. In principle, *ψ* rises with *z_l_*. However, the influence of the thickness of the liquid layer on the dose rate is negligible when *z_l_* ≪ *λ*_IMFP_, which refers to only a single inelastic scattering event along the trajectory of an electron through the sample.

Finally, we emphasize that the dose rate is sensitive to the acceleration voltage. The inelastic mean free path of electrons in water is shortest around an electron energy of only 0.1 keV.^[^ ^53^ ^]^ Hence, the probability of inelastic electron‐water interactions and, thus, the stopping power, decreases for higher acceleration voltages. As a consequence, dose rates experienced by water in typical scanning electron microscope configurations (SEMs) can greatly exceed those achieved in (typical) TEM setups: For a 100 nm thin liquid layer, the dose rate is ≈14, 100, or 1 000 times higher when irradiated with 20, 5, or 1 kV instead of 300 kV, assuming values for *S* and *λ*_IMFP_ at 1, 5, and 20 kV following ref. [69] and neglecting the energy loss of the primary beam.

## 3 Radiation Chemistry in Liquid Phase Electron Microscopy

In this section, we start to dive into the consequences of inelastic scattering in terms of measurable artifacts. We do so by discussing the most prominent beam effect during LP‐TEM, which is radiolysis of the liquid phase by ionizing radiation. While we illustrate these effects with electron irradiation in LP‐TEM, we note that these concepts apply more generally to low‐LET irradiation other than electrons, as discussed in Section 2.

**Figure 2:**
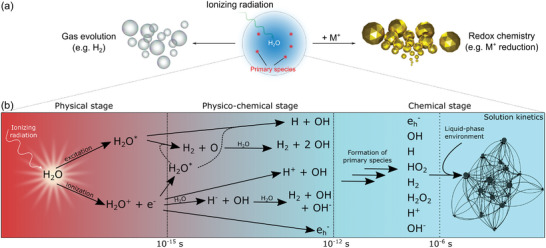
a) Radiolysis of water can trigger phase transitions that yield bubbles or particle nucleation. b) This results from a physicochemical relaxation cascade that yields reactive chemical species. Panel (b) is reproduced from ref. [70], under a CC BY 4.0 license.

Radiolysis can result in phase transitions such as gas bubble formation or particle precipitation of a metal precursor solution (**Figure** **2**a).^[^ ^71^ ^]^ This is related to an energy transfer from the probe to the specimen. Exposure of matter to high‐energy radiation causes the ionization of material components and, thus, the fission of chemical bonds (radiolysis). Subsequently, reactive fragments undergo reactions with surrounding substances (radiation chemistry). Examples of irradiation sources that cause these phenomena are high‐energy photons (i.e., UV photons, X‐rays, or γ‐rays), protons, heavy ions (e.g., helium ions), and electrons. The fundamentals of radiation chemistry are elucidated in depth elsewhere. We focus this discussion on electron irradiation of systems relevant to liquid phase electron microscopy, most crucially, the inelastic interactions between the electron beam and water.

### 3.1 Radiation Chemistry of Water

Water radiolysis is, understandably, a well‐studied process. Here, we summarize the fundamental principles based on refs. [55, 72, 73, 74], to which the interested reader is referred for more details.

The underlying mechanism can be described as a relaxation cascade, depicted in Figure 2b. The primary excitation of water occurs within the trajectory of the electron as it traverses the specimen. These excitations occur randomly alongside the electron pathway through the liquid layer, with a statistically averaged distance that matches the inelastic mean free path of electrons in water. The excitation products subsequently react and diffuse, forming spherically expanding volumes with altered composition, known as spurs.^[^ ^71^ ^]^ Within a spur, inelastic scattering first causes excitation and ionization of H_2_O (the physical stage). After ≈1 fs, the formed species relax via physicochemical processes, yielding reaction products that are concentrated inhomogeneously within the spur after ≈1 ps (the physico‐chemical stage). These products are referred to as the initial yield of radiolysis. Considerations based on spatial distributions of the initial yields reveal a spur size of a few nm.^[^ ^75^, ^76^ ^]^

During these stages, the excited species diffuse so the spurs broaden. After ≈0.1–1 µs,^[^ ^73^, ^74^ ^]^ the concentration profiles evolve toward a homogeneously distributed set of primary products that mainly react chemically. The primary products are listed in Equation (4):

(4) $$H_{2}O\overset{\mathrm{Ionizing}\;\mathrm{radiation}}{\to }H^{+},\;{\mathrm{OH}}^{-},\;\mathrm{OH},\;H,\;{\mathrm{HO}}_{2},H_{2}O_{2},H_{2},\;e_{h}^{-}$$

Here, e_h_^−^ is the solvated electron. These species eventually react with each other and with their environment, as indicated by the last part of Figure 2b. The consequences arising from these solution kinetics are discussed below:

The formation rate of these primary products is summarized in so‐called generation values (*G*‐values, *G*_i_), which refers to the number of each species generated per absorbed energy per time (dose rate). *G*‐values are historically denoted in units of molecules per 100 eV, which translates to $\frac{\mathrm{mol}}{J}$ in SI units. To date, no experimental assessment of *G*‐values under LP‐TEM conditions has been reported. Hence, quantification for LP‐TEM relies either on Monte‐Carlo simulations^[^ ^77^, ^78^ ^]^ or experimental measurements under related conditions. As γ‐radiation shows energy transfer characteristics similar to those of high‐energy electrons, measurements with γ‐radiation have been used as an alternative.^[^ ^78^, ^79^, ^80^ ^]^ Some modeled and experimental values are listed in **Table** **1**. However, both *G*‐value sets shown here are initially obtained for low dose rates, so they are only reliable as long as spurs remain well separated.^[^ ^71^ ^]^ In practice, the lack of an alternative leads LP‐TEM practitioners to extrapolate lower dose rate data to high dose rates.

**Table 1:** *G*‐Values that may be suitable for LP‐TEM. Hill & Smith^[^ ^77^ ^]^ simulated values which are extracted and atom‐balanced by Schneider et al.^[^ ^78^ ^]^ for high‐energy electrons (200–300 kV). Pastina and LaVerne^[^ ^79^ ^]^ measured *G*‐values for γ‐radiation.

Reactant	*G*_i_ after Hill and Smith^[^ ^77^, ^78^ ^]^		*G*_i_ after Pastina and LaVerne^[^ ^79^ ^]^	
	[molecules / 100 eV]	[10^−7^ mol J⁻^1^]	[molecules / 100 eV]	[10^−7^ mol J⁻^1^]
e_h_^−^	3.47	3.60	2.60	2.69
H^+^	4.42	4.58	3.10	3.21
OH^−^	0.95	0.98	0.50	0.52
H_2_O_2_	0.47	0.49	0.70	0.73
H	1.00	1.04	0.66	0.68
OH	3.63	3.76	2.70	2.80
HO_2_	0.08	0.08	0.02	0.02
H_2_	0.17	0.18	0.45	0.47
H_2_O	−5.68	−5.89	−4.64	−4.81

We note that Equation (4) allows for building kinetic models that describe the environment in the chemical stage, which we will discuss in Section 6. As *G*‐values summarize the influence of the beam, the subsequent chemistry is independent of the source of irradiation, enabling the usage of reaction rate constants measured in adjacent conditions.^[^ ^81^ ^]^

### 3.2 Implications for Liquid‐Phase Electron Microscopy

Subsequent reactions of the primary species formed in water quickly span a reaction network of up to 17 reactants.^[^ ^78^, ^82^ ^]^ In this section, we start by describing the chemical environment of pure water irradiated in LP‐TEM. We move on to aqueous solutions in Section 3.3.

**Figure 3:**
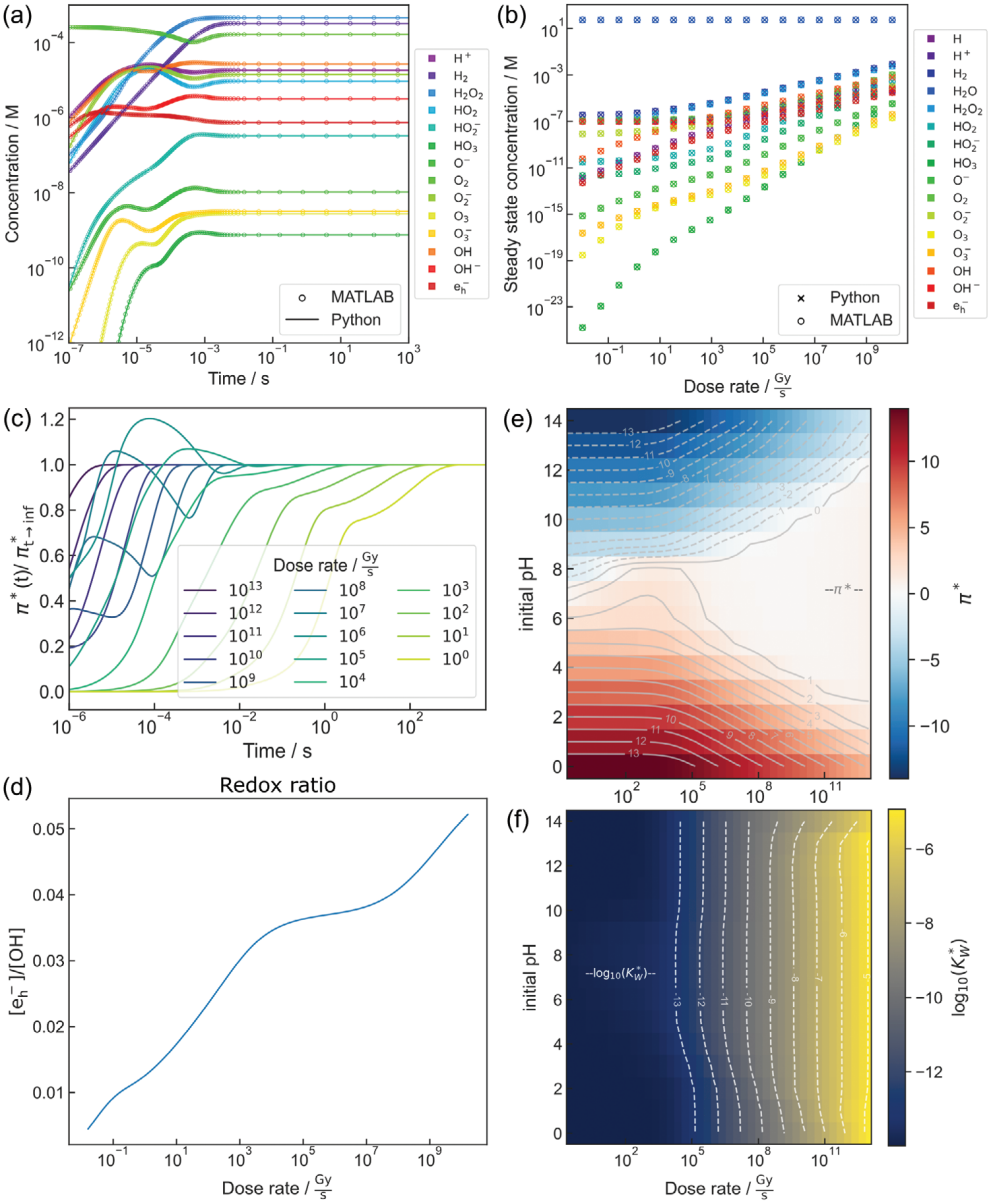
Radiation chemistry of water differs from beaker chemistry conditions due to the formation of radiolysis products: a) shows the temporal evolution of radiolysis products and formation of a steady state for a dose rate of 72 MGy s⁻^1^ (reproduced from ref. [83], using a model introduced in ref. [78]). Dose rate‐dependent steady‐state evolution (b) shows that the magnitude of radiolysis effects scales with dose rate. Moreover, (a) and (b) show concentration changes independent of the modeling platform. (a) and (b) are reproduced (legend adjusted) from ref. [83]. c) Temporal evolution of steady state formation as indicated by the transient of the radiolytic acidity (π*) toward its steady state value for different dose rates at an initial pH value of 7. The transient duration decreases with dose rate. From ref. [70]. d) A ratio plot of the most important redox species in irradiated, deaerated water (pH 7) shows that the redox conditions can differ with dose rate. Reproduced from ref. [78]. The radiolytic acidity (e) as a function of dose rate shows that the ratio of H^+^ and OH^−^ (almost) equalizes with the dose rate, even under different initial pH values. The radiolytic ion product (f) scales with dose rate, while mainly unaffected by the initial pH. (e) and (f) are reproduced from ref. [70]. (a–c), (e), and (f) are published under a CC‐ BY 4.0 license.

Under continuous supply of primary products (e.g., due to TEM imaging), the species concentrations adjust themselves until recombination reactions counterbalance the generation rate of each species. This prevents water depletion below ≈10^13^ $\frac{\mathrm{Gy}}{s}$ ^[^ ^78^ ^]^ and causes the formation of dose rate‐dependent steady states. A steady state characterizes a dynamic equilibrium toward which the system relaxes as long as irradiation continues. We illustrate this in **Figure** **3**a using a kinetic model of the radiation chemistry of pure, deaerated water at 72 $\frac{\mathrm{MGy}}{s}$.^[^ ^78^ ^]^ To select the parameters for this model, we note that Section 2 includes a discussion on LP‐TEM‐relevant dose rates, and the modeling details are described in Section 6.1. Figure 3a shows that radiolytic steady‐state conditions (Figure 3b) can build up within milliseconds at dose rates relevant to typical LP‐TEM experiments (when disregarding diffusion).^[^ ^78^ ^]^ For dose rates below about 10^3^ $\frac{\mathrm{Gy}}{s}$, the steady state builds up more slowly, as indicated in Figure 3c by the evolution of radiolytic acidity, a quantity discussed below.

Radiolytic steady‐state conditions during LP‐TEM differ substantially from non‐irradiated benchtop chemistry. Most prominently, the simultaneous generation of strong oxidants and reductants triggers redox processes.^[^ ^71^, ^78^ ^]^ In particular, e_h_^−^ and H exhibit highly reductive standard potentials relative to the normal hydrogen electrode *E*° (*E*°(H_2_O/e_h_^−^) = −2.88 ± 0.02 V and *E*°(H^+^/H) = −2.31 ± 0.03 V), which are opposed by oxidative OH radicals (*E*°(OH/H_2_O) = +2.730 ± 0.017 V).^[^ ^84^ ^]^ The evolution of the concentration ratio of the two most aggressive redox‐relevant species is shown in Figure 3d.

Similarly, radiation affects acid‐base chemistry. As both H^+^ and OH^−^ are primary species, both are generated upon electron irradiation of water. This has two key consequences: First, charge balance demands a higher H^+^ than OH^−^ concentration at low dose rates because H^+^ compensates for all anions generated by radiation chemistry (mainly OH^−^, O_2_^−^, HO_2_^−^, and e_h_^−^). This yields a (slight) drop in pH. This must not be confused with a transient pH change at ultrashort timescales during spur evolution.^[^ ^85^ ^]^ Second, when the dose rate exceeds about 1000 $\frac{\mathrm{Gy}}{s}$, simulations predict a monotonic increase in the steady‐state concentration of both species. This can be interpreted as the generation rate and the subsequent chemical reactions bypassing the otherwise predominant autoprotolysis equilibrium of water. An important consequence is that the concept of pH loses much of its applicability because H^+^ and OH^−^ concentrations are not directly coupled anymore.^[^ ^70^ ^]^

To gauge the acidity under irradiation, both species must be considered. This can be performed by calculating the radiolytic acidity π* and the radiolytic ion product $K_{W}^{*}$:^[^ ^70^ ^]^

(5) $$\pi^{*}=\;\mathrm{lg}\left(\frac{\left[H^{+}\right]}{\left[OH^{-}\right]}\right)$$

(6) $$K_{W}^{*}={\left(\left[H^{+}\right]\cdot \left[OH^{-}\right]\right)}_{\mathrm{irradiated}}$$

Mapping π* for different (initial) pH and dose rates reveals that irradiation of water drives the system toward neutral and (slightly) acidic conditions, Figure 3d, while $K_{W}^{*}$ rises almost linearly with the dose rate, Figure 3f. This translates to enormously elevated H^+^ and OH^−^ steady‐state concentrations. Therefore, when a specimen is exposed to irradiated water, its reactivity with both ions must be considered.^[^ ^70^ ^]^

The discussion above suggests that thermodynamic consequences of irradiation should be considered in any analysis of LP‐TEM data. Under the assumption that every infinitesimal step of the reaction cascade can be approximated as, in principle, reversible, this can be regarded as a shift in the Gibbs free energy landscape. In particular, radiation is considered as a change in chemical potential Δ*µ*. When forming a new phase (e.g., a particle or bubble), this can be expressed by:^[^ ^83^ ^]^

(7) $$\Delta \mu =k_{B}T\ln\left(\frac{c_{0}}{c_{\mathrm{steady}\text{--}\mathrm{state}}}\right)$$

Here, *k*_B_ is the Boltzmann constant, *T* the temperature, *c*_0_ the initial and *c*_steady‐state_ the corresponding steady‐state concentration of the heterophase‐relevant species. On top of this, irradiation can also cause heating or the build‐up of electric fields (membrane charging), as we will discuss below in Sections 4.1 and 5. This changes the Gibbs free energy landscape further. Thus, irradiation affects whether a process occurs spontaneously or not.

### 3.3 Beyond Pure Water: The Approximation of Diluted Solutions

Water is the basis of most LP‐TEM experiments and so is its radiation chemistry. Yet, extrapolating findings valid for pure water to aqueous solutions can become a false friend and must be validated before use. Additives, contaminants, or dissolved species have implications for redox chemistry, acid‐base chemistry, and gaseous products. We will first emphasize this through three types of experimental evidence and subsequently discuss the modeled radiation chemistry of such systems.

**Figure 4:**
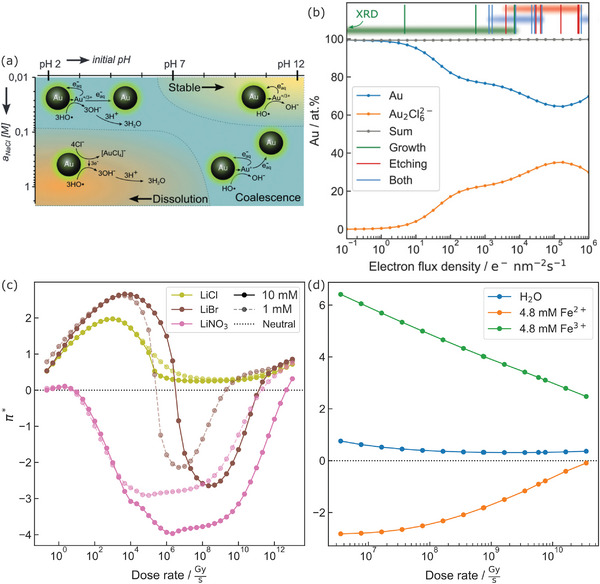
a) Schematics of gold nanoparticle reactivity under varying Cl^−^ activities *a* and initial pH conditions in LP‐TEM, showing that gold reactivity increases with chlorine activity and decreasing pH. Note that the reaction pathways are simplified by disregarding the role of chlorine species. Used with permission of The Royal Society of Chemistry from ref. [86], permission conveyed through Copyright Clearance Center, Inc. copyright The Royal Society of Chemistry 2015. b) Simulated steady‐state evolution of gold species in 20 mm HAuCl_4_ solution showing reduced stability of Au, accompanied by experimental classifications, from ref. [83]. c,d) Radiolytic acidity for aqueous solutions comprising ionic compounds. c) shows 10 (bold) and 1 (pale, dashed) mm LiCl (the data of aerated water coincides with 1 mm LiCl), LiBr, and LiNO_3_. Reproduced from ref. [70]. d) shows solutions containing Fe^2+^ or Fe^3+^ with water plotted for comparison. From ref. [88]. Panels (b), (c), and (d) are published under a CC‐BY 4.0 license.

Experiments involving solutions show redox phenomena that are not explicable with pure water alone. For instance, even the redox chemistry of noble metal nanostructures, such as gold, heavily depends on the additives within the aqueous solution: Several studies confirmed that oxidative etching of gold nanoparticles is only pronounced at both high chlorine and high (initial) H^+^ concentrations, whereas low concentrations favor growth or agglomeration^[^ ^83^, ^86^, ^87^ ^]^ (**Figure** **4**a,b). The dose rate‐dependent radiation chemistry of pure water alone does not capture such solute effects.

Similarly, acid‐base chemistry behavior is solute‐dependent. Irradiation of CeNO_3_ solutions can yield cerium hydroxide precipitation, suggesting elevated OH^−^ concentrations.^[^ ^89^ ^]^ In contrast, the beam‐induced etching of iron oxide minerals follows a mechanism known from benchtop chemistry as acidic dissolution.^[^ ^88^, ^90^ ^]^ Pure water simulations predict simultaneously elevated concentrations of H^+^ and OH^−^.^[^ ^70^ ^]^ Either experimental observation can point toward system‐specific reactivities with H^+^ and OH^−^, but as we will discuss below, the corresponding radiolytic acidity suggests a different explanation.

As a third example, while the radiation chemistry of pure water predicts the formation of gaseous hydrogen bubbles well,^[^ ^71^, ^78^, ^91^ ^]^ the chemistry in the vicinity of radiolytically formed nanobubbles in LP‐TEM does not always match the expected chemical behavior of H_2_. Despite reports of bubbles shielding nanostructures from etching (as expected for H_2_ in these cases),^[^ ^92^, ^93^ ^]^ different conditions trigger the formation of oxidative gaseous phases.^[^ ^83^, ^94^, ^95^ ^]^ This requires a change in bubble composition, e.g., due to the presence of volatile radiolysis (by)products.

**Figure 5:**
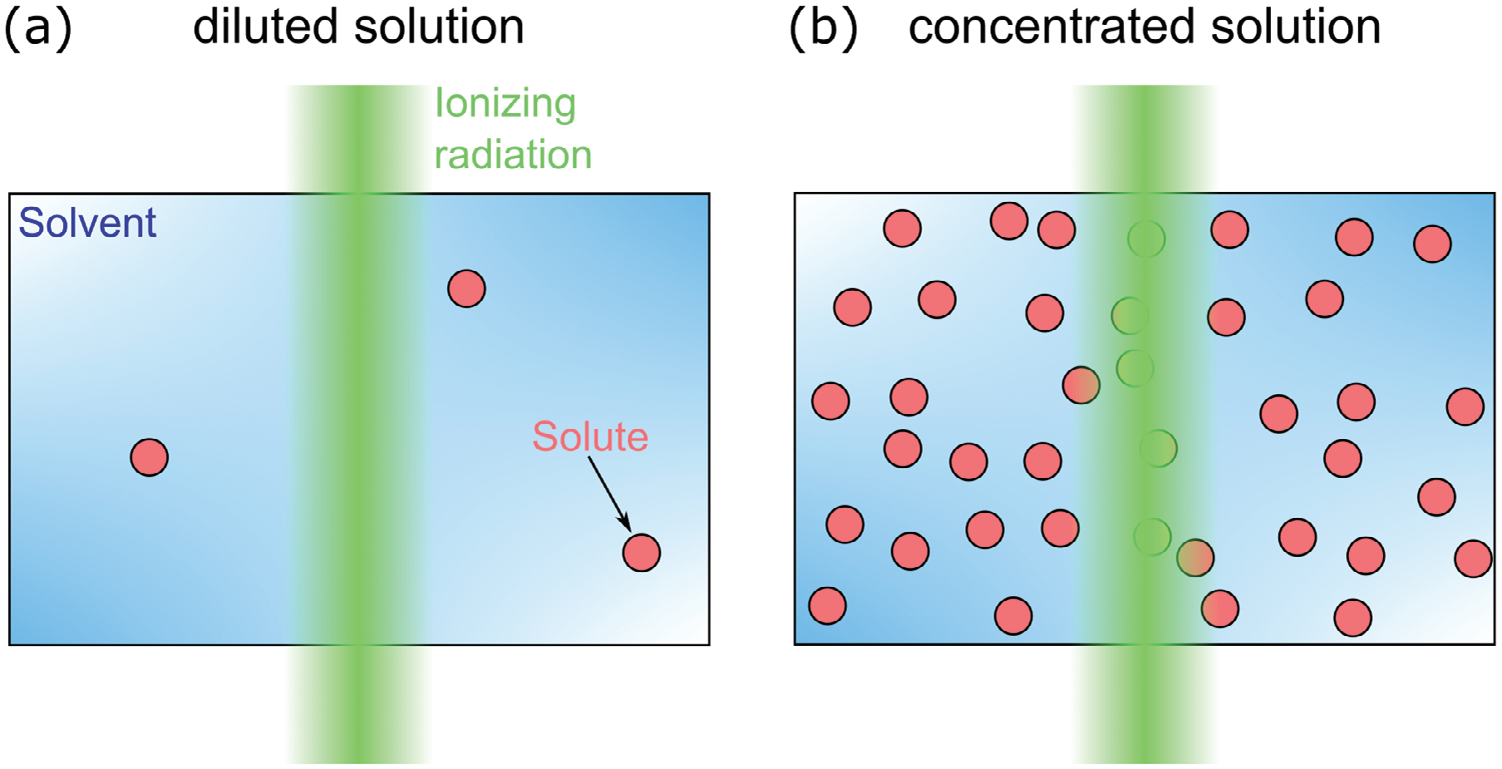
Illustration of diluted and non‐diluted irradiated solutions. a) For diluted solutions, it is sufficient to assume that ionizing irradiation (green) only reacts with solvent molecules (blue background). b) At high concentrations, solutes (red circles) cannot be neglected for *G*‐value formation.

To account for solute effects such as in these three examples, we need to consider the interplay of radiation chemistry with solvated compounds. We distinguish two cases depending on solute concentration. For highly diluted solutions, ionizing radiation is assumed only to interact with the solvent so that spur relaxation remains unaffected by solutes (**Figure** **5**a). This dilution approximation does not hold at high solute concentrations (Figure 5b), so additional beam‐induced physical and physico‐chemical interactions must be considered. Within the radiation chemistry community, the concentration limit for applicability of the dilution approximation is taken to be in the range of 0.1^[^ ^73^ ^]^ to 1 m.^[^ ^74^ ^]^ We will first discuss applications of the dilution approximation, and second, we will discuss non‐diluted systems in LP‐TEM.

#### 3.3.1 Redox Chemistry in Solutions: Exemplary Discussion on a Three‐Cluster‐System

Meaningful kinetic models require accurate inclusion of the interplay between the reactants present in irradiated water and the solutes, and also between different, closely related groups of reactants that are connected by chemical reactions (reactant cluster). Every disregarded reactant cluster can potentially cause a divergence between the model and experiment, creating possible pitfalls for interpretation. The case of diluted aqueous HAuCl_4_ solutions provides a good illustration.

Aqueous HAuCl_4_ solutions are one of the most studied model systems for LP‐TEM. Not only is HAuCl_4_ an interesting precursor for gold nanostructures, but its vivid redox chemistry and the high contrast of gold nanostructures in TEM make it a frequent candidate for LP‐TEM training. Nonetheless, its redox radiation chemistry remained a mystery for about a decade.

The first attempt to augment the pure water model toward describing diluted, aqueous HAuCl_4_ solutions incorporated chlorine reactants, as they dominate the radiation chemistry of aqueous HCl and NaCl solutions.^[^ ^96^ ^]^ The modeling predicted a reduction of highly reactive radicals during steady‐state formation, suggesting a scavenging effect by chlorine in LP‐TEM. This was supported by subsequent studies^[^ ^97^ ^]^ and the underlying scavenging mechanism is explored in Section 3.6. Yet, this first attempt does not predict the behavior of the gold. Likewise, the incorporation of a gold reduction cascade from Au^III^ to Au^0^ in the pure water model for LP‐TEM^[^ ^80^, ^98^ ^]^ does not elucidate the dependence of gold nanoparticle evolution in LP‐TEM on the chlorine concentration.

Only by combining the gold and chlorine clusters was the radiation chemistry of HAuCl_4_ solutions explained and an agreement in steady‐state evolution of Au^(0)^ between experiment and simulation achieved (Figure 4b).^[^ ^83^ ^]^ The radiation chemistry of HAuCl_4_ requires taking both groups of reactants into account because key oxidative and reductive processes occur via different cluster interactions. Gold reduction is achieved via primary products (e_h_^−^ and H‐radicals) while etching occurs by a secondary, chlorine‐containing species.^[^ ^83^, ^96^ ^]^ As byproducts, the full simulation suggests high concentrations of volatile species such as molecular HCl and chloro‐gold(II) complexes. This explains the observed corrosive gas phases and gold transport via gas bubbles.^[^ ^83^, ^94^ ^]^

This example illustrates two aspects of LP‐TEM: the necessity of considering all reactant clusters, as the main reaction pathways can shift depending on the boundary conditions, and the predictive power of radiation chemistry modeling if the system is pictured appropriately.

#### 3.3.2 Solute Effects on the Radiolytic Acidity

Solutions containing salts also show a redox interplay that can cause a redistribution of charges between the chemical reactants, including H^+^ and OH^−^. The radiolytic acidity at a given dose rate strongly depends on the actual composition of the solution. This is shown in Figure 4.

Here, LiCl solution appears from modeling to yield a slightly acidic solution similar to that of water, while LiNO_3_ solutions are expected to cause alkaline conditions with a concentration‐dependent magnitude. As shown for LiBr, the solution may even change its behavior between acidic and alkaline as a function of dose rate and solute concentration in the typical dose rates relevant for LP‐TEM, Figure 4c.^[^ ^70^ ^]^ Similarly, Fe‐containing solutions are shown to yield oxidation‐state dependent radiolytic acidity (alkaline for Fe^2+^, and acidic for Fe^3+^, Figure 4d).^[^ ^88^ ^]^ The results shown in Figure 4c also suggest that the radiolytic acidity converges to the values in irradiated water at high dose rates.

These calculations suggest that radiation chemistry can be harnessed to tailor a desired acidity, to a certain extent, by employing salt additives. It is worth pointing out that this is accompanied by only minor changes in $K_{W}^{*}$, whose order of magnitude remains mainly dose‐rate controlled.

#### 3.3.3 Non‐Diluted Solutions

If the concentrations of solutes become large, the assumption that ionizing radiation solely interacts with the solvent no longer holds (Figure 5b). Consequently, *G*‐values based on the dilution approximation do not describe the system well.^[^ ^99^ ^]^ Instead, the initial interaction and the subsequent relaxation cascade must be adjusted, with *G*‐values becoming a function of the solute concentrations. In practical terms, *G*‐values may change substantially during an experiment if the solute concentrations are altered.

### 3.4 Organic Solvents

Although the majority of radiolysis studies in LP‐TEM experiments target aqueous systems, LP‐TEM experiments in organic solvents are of great interest as a platform to understand solution‐phase soft nanomaterials dynamics, including self‐assembly, nanostructure formation, and polymerization.^[^ ^11^, ^100^ ^]^ In organic materials and solvents, reactive species from electron beam‐induced radiolysis are also expected to affect the chemical environment.^[^ ^73^ ^]^ As in water, this may lead to phenomena such as nucleation, growth, and crystallization.^[^ ^101^, ^102^, ^103^ ^]^ It is, therefore, crucial to address radiation chemistry and understand the effect of electron scattering in organic materials. However, radiolysis in organic solvents is less understood for LP‐TEM conditions. Given the variety of organic liquids, we aim to provide an illustrative picture of recent findings in LP‐TEM and general guidelines based on the experience of the radiation chemistry community.

As is the case for water, the initial interaction of ionizing radiation with an organic compound R causes the formation of solvated electrons (due to the lack of a hydrate shell, these are herein denoted as e^−^_s_) and partly cationic radicals that denoted for simplicity as R ^· / +^:^[^ ^73^ ^]^

(8) $$R\overset{\mathrm{Ionizing}\;\mathrm{radiation}}{\to }R^{\cdot /+}+{e_{s}}^{-}$$

Analogously to water, recombination and subsequent reactions with the solvent form a reaction cascade that can be summarized by primary yields (*G*‐values). Although solvent‐specific, a few key factors and trends can be stated. One of these is that the polarity of the solvent increases the yield of free ions that escape recombination (*G*_fi_). Furthermore, in nonpolar solvents, the size and shape of the molecules appear to influence *G*_fi_, resulting in an increase in large and spherical structures such as neopentane. In contrast to water, irradiation‐induced polymerization and cross‐linking is a frequent consequence that needs to be considered.^[^ ^73^ ^]^ Heteroatoms can alter radiation chemistry further.^[^ ^104^ ^]^

The number of unsaturated C‐C bonds also plays a crucial role. In particular, aromatic hydrocarbons are specifically dose‐resistant. They can tolerate high‐energy electron irradiation because their π‐electron system delocalizes the excitation energy, hampering bond breakage. Consequently, the production of free radicals is limited due to the electron‐ion recombination process.^[^ ^105^ ^]^ This is illustrated by comparing *G*‐values of cyclohexane and benzene. In cyclohexane, the primary H_2_ yield is 0.58 µmol J^−1^, but this decreases to ≈0.004 µmol J^−1^ in benzene. *G*_fi_ values range on the order of 0.015 µmol J^−1^ for cyclohexane and 0.005 µmol J^−1^ for benzene.^[^ ^73^ ^]^

**Figure 6:**
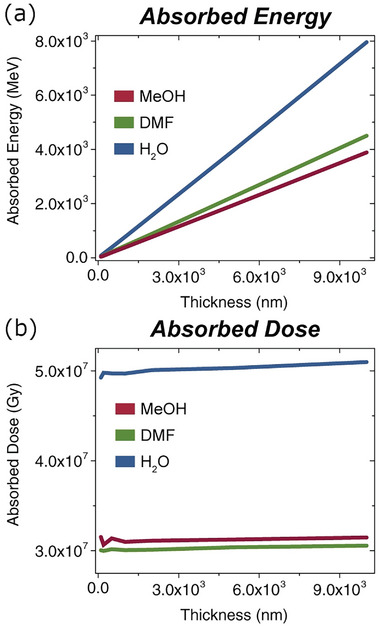
Monte Carlo simulation results under conditions representative for LP‐TEM using methanol (MeOH), DMF, and water, showing a) absorbed energy and b) experienced dose. In both cases, water absorbs energy more strongly than organic solvents. Reproduced from ref. [106], with permission from Elsevier. Copyright 2022, Elsevier.

For more comprehensive information, Monte Carlo simulations of spur evolution‐ are used to obtain *G*‐values. Combined with reaction kinetics, this yielded the first models of the radiolysis of dimethylformamide (DMF),^[^ ^106^ ^]^ methanol,^[^ ^106^ ^]^ and isopropanol^[^ ^107^ ^]^ for LP‐TEM. A literature survey of *G*‐values for isopropanol and toluene can be found in ref. [108]. Compared to water, organic liquids have a lower density and oxygen percentage, and therefore absorb less energy and experience a lower dose than their aqueous equivalents, as shown in the examples of DMF and methanol in **Figure** **6**a,b. Consequently, beam‐sensitive materials have been found to be more stable in DMF than in water.^[^ ^109^ ^]^ Similarly, radiation chemistry in isopropanol leads to less reactive radicals with lower steady‐state concentrations than their aqueous counterparts.^[^ ^107^ ^]^ Therefore, LP‐TEM based on organic solvents can be advantageous in imaging low‐contrast solvated organic materials that require deliberate dose monitoring, or in studying inorganic nanocrystal evolution with reduced radiolysis effects.^[^ ^108^ ^]^

The radiolytic sensitivity of organic molecules plays a vital role in polymer synthesis by triggering polymerization reactions. Electron beam‐induced radical processes are gaining interest for polymer synthesis because of direct radical generation from the solvent and controllable electron penetration depths. In this context, LP‐TEM could serve as an effective tool to study polymerization‐induced self‐assembly by capturing the formation of spherical assemblies induced by polymerization.^[^ ^110^ ^]^ However, applying the knowledge obtained from LP‐TEM to industrial‐scale polymer synthesis using electron irradiation requires the quantification of the organic radiation chemistry in LP‐TEM.^[^ ^111^ ^]^

Even though organic solvents are more resistant to high‐energy electron irradiation than water, radiolysis must still be considered in LP‐TEM experiments that involve these solvents as it can drastically change the chemical environment. Although systematic assessments of the radiation chemistry of organic liquids in LP‐TEM are becoming more widespread, they are still not universal. Nonetheless, further specifics on the radiation chemistry of individual organic liquids have been provided in the radiation chemistry community.^[^ ^73^, ^104^, ^112^, ^113^, ^114^, ^115^ ^]^ Understanding radiation chemistry in organic solvents is a critical step toward correctly interpreting nanostructure formation in organic media based on LP‐TEM data.

### 3.5 Diffusion, Parallel Illumination, and Scanned Probes

The initiation of radiation chemistry during electron microscopy implies that the shape of the probe itself is important. Unsurprisingly, parallel beam (TEM) and scanning beam (STEM) modes can exhibit different outcomes in terms of radiolysis.^[^ ^116^, ^117^ ^]^ This is partially related to diffusion and therefore additionally affected by the geometry of the LC. We will only briefly discuss the consequences of LC geometries on radiolysis and focus on general concepts of irradiation mode and diffusion regarding radiation chemistry.

**Figure 7:**
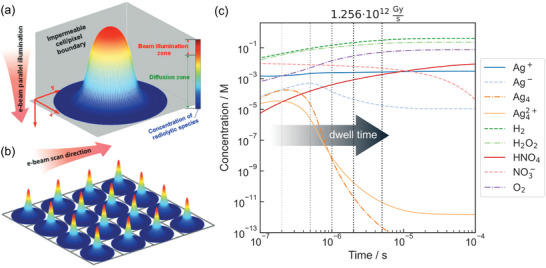
Schematic concentration profiles of radiolysis products under a) TEM and b) STEM irradiation modes. While TEM uses a broad beam with notable differences in concentration inside the irradiated volume, STEM causes several, locally confined radiolysis spots that interact diffusively. The irradiated cross‐section is denoted in red. Reproduced from ref. [81]; CC‐BY 3.0 licensed. c) Temporal evolution of a 10 mm AgNO_3_ solution irradiated with a STEM‐beam dose rate, showing the main products and species found relevant to Ag growth and nucleation.^[^ ^119^ ^]^ The vertical lines denote typical dwell times which are located within the transient of the simulation, causing a different radiation chemistry. Reproduced after ref. [120].

Under parallel illumination (TEM), the primary electron energy is distributed homogeneously and continuously. If the total liquid volume is irradiated, such as in graphene liquid pockets smaller than the beam diameter, the radiolytic species are homogeneously distributed and diffusion can be neglected. Consequently, the concentrations of the radiolysis products evolve continuously until a steady state is reached, as explored in Section 3.2. If the liquid is only partially irradiated, concentration gradients drive the diffusion of radiolysis products out and precursor species of the initial solution into the irradiated volume. The magnitude of these gradients depends on the dose rate, the beam diameter, the position of the beam in the LC, and the chosen geometry of the LC. Larger beam diameters (TEM) and smaller cell volumes cause faster built‐up of radiolytic species,^[^ ^81^ ^]^ whereas LC geometries can be designed to provoke steep gradients for rapid solution exchange^[^ ^118^ ^]^ or to suppress diffusion as in homogeneously irradiated graphene liquid pockets. The system still converges toward a steady state with homogeneous species distribution; however, this can take orders of magnitude longer than in the homogeneous case if the whole cell is regarded. Nonetheless, the concentrations in the center of the irradiated volume converge toward their steady state value almost as rapidly as in the homogeneous case.^[^ ^55^ ^]^ Under partial irradiation of the liquid volume, the concentration of species in the beam still depends on geometry. **Figure** **7**a illustrates this schematically, showing a concentration decrease from the optical axis (dark red) to the cell boundary.

In STEM, on the contrary, the liquid is exposed to irradiation only during the restricted period the scanning beam remains stationary (dwell time) and in a very limited volume, with parameters based on the scan parameters of the probe. This is illustrated in Figure 7b. Meanwhile, due to the focused beam, orders of magnitude higher dose rates are delivered for typical experimental parameters. Therefore, more violent radiolysis is expected which is accompanied by steep concentration gradients caused by the small probe diameter. Nonetheless, beam damage in TEM appears to spread further into the non‐irradiated volume than in STEM.^[^ ^109^ ^]^ We discuss possible explanations in the following paragraphs.

Typical in situ STEM dwell times range in µs, which can be insufficient to reach a steady state, even at high dose rates. This is demonstrated for an AgNO_3_ solution in Figure 7c, in which the solution chemistry depends on the dwell time.^[^ ^120^ ^]^ Evidently, stable radiolysis products with comparably long diffusion ranges are still building up during STEM irradiation. This simple example assumes a perfectly mixed solution and, therefore, neglects diffusion. As diffusion slows down the build‐up of steady states, this trend will be more pronounced when diffusion is incorporated.

For small interpixel distances during STEM imaging, single spur events of neighboring pixels may overlap, thus changing the primary product distribution. Self‐scavenging reactions would reduce the amount of radical species in favor of forming less reactive molecular products such as H_2_ or H_2_O_2_.^[^ ^71^ ^]^

Even without spur overlap, diffusion between different irradiated positions in STEM can influence the resulting radiation chemistry. Such an interpixel diffusion can increase the concentration of radiolytic products. Intuitively, this effect is reduced by increasing the separation distance between individual beam locations via the STEM magnification. Depending on the lifetime and diffusivity, this effect might be selective for certain species. Following this logic, increasing the distance between consecutively irradiated pixels by changing the scanning pattern could mitigate the cross‐talk effect between individual diffusion zones. A higher step separation and especially sub‐sampling leads to a lower average concentration of radiolytic products. A longer dwell time, on the other hand, leaves more time for radiolytic processes and therefore in general also increases the concentration of radiolysis products.^[^ ^81^ ^]^ Overall, we expect differences in radiolysis phenomena between TEM and STEM at nominally similar electron flux densities, and this should be considered when planning or comparing experiments.

### 3.6 Radical Scavenging for Gaining Control of Radiation Chemistry

Using additives to mitigate the influence of radical chemistry during LP‐TEM is a powerful approach to gaining control of some aspects of LP‐TEM experiments. Scavenging relies on the conversion of highly reactive radicals to products with a smaller redox potential with regard to the desired outcome. Due to the high reactivity of e_h_^−^, H, or OH radicals, this is usually the case when adding solutes to aqueous solutions. However, reactions of radicals with non‐radical scavenger molecules usually produce secondary radicals, which may alter the environment themselves.^[^ ^121^ ^]^ For successful scavenging, those should be inert compared to the reactant.^[^ ^73^ ^]^

One general approach that has been successfully transferred to LP‐TEM involves the scavenging of OH and H by organic solutes.^[^ ^90^, ^98^, ^117^, ^122^ ^]^ For instance, radiolytic polymer damage is reduced by employing isopropanol for scavenging.^[^ ^98^ ^]^

**Figure 8:**
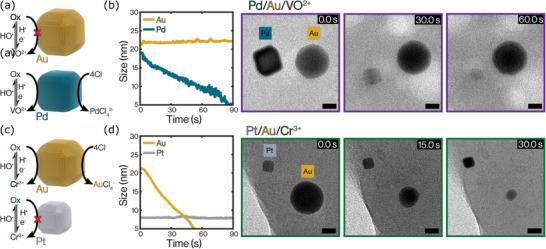
The presence of different redox couples facilitates materials‐specific etching by electron beam irradiation: a) schematics and b) LP‐TEM evidence that an irradiated V(IV) solution facilitates selective Pd etching while Au nanostructures remain constant, and c) schematics and d) LP‐TEM evidence that a Cr(III)‐containing suspension with Au and Pt nanoparticles causes selective Au etching. The scale bars are 10 nm. Reproduced with permission from ref. [123], Copyright 2021, American Chemical Society.

In another approach, additives are used to control the reactivity of solutions and provide defined redox potentials within LP‐TEM. If additives provide redox couples that quickly react with primary species, the products have a smaller redox potential than the primary radicals. This can provide a distinct selectivity so that these equilibria can be used to tailor specific redox environments. **Figure** **8** illustrates this concept using different noble redox couples to provide opportunities for controlled materials etching. For Pt, Au, and Pd nanoparticles in graphene LCs, adding a VO^2+^/VO_2_^+^ redox environment (Figure 8a) etches Pd but not Au (Figure 8b). In turn, the Cr^3+^/HCrO_4_^+^ couple (Figure 8c) etches Au but not Pt nanoparticles (Figure 8d). This facilitates materials‐specific redox studies and can be interpreted as a redox equivalent to pH buffer solutions.^[^ ^123^ ^]^

Radiation chemistry experience suggests further strategies to supply tailored chemical conversion platforms. For instance, saturating aqueous solutions with N_2_O eventually converts e_h_^−^ into OH, thus converting reducing into oxidizing conditions within nanoseconds:^[^ ^73^ ^]^

(9) $$e_{h}^{-}+N_{2}O\to N_{2}+O^{-}$$

(10) $$O^{-}+H_{2}O\to \mathrm{OH}+OH^{-}$$

A compilation of empirical examples including those intended to provide fully reducing conditions is provided by ref. [73]. However, as pulse radiolysis studies are usually performed at low dose rates, care must be taken when transferring this knowledge to LP‐TEM experiments. Hence, the challenge remains to maintain a holistic understanding of such systems under irradiation, including influences of new reaction routes introduced by the scavenger. Its use should be balanced with the network of chemical reactions in radiation chemistry to obtain the desired scavenging effect.

#### 3.6.1 Graphene Membranes as Radical Scavengers

Membrane chemistry is known to affect LP‐TEM observations, both through charging and chemical effects. An example of this is the observation that DNA‐separated gold nanoparticle superlattices are significantly more durable when encapsulated in graphene LCs compared to those with silicon nitride windows.^[^ ^124^ ^]^ This is attributed to graphene‐induced suppression of OH‐mediated oxidative DNA degradation via sacrificial reactions of the graphene with OH radicals, forming graphene oxide.^[^ ^125^, ^126^ ^]^ Reduced radiation damage has been verified for other biological^[^ ^127^, ^128^ ^]^ and soft matter^[^ ^129^, ^130^ ^]^ samples coated with graphene. Moreover, Raman measurements demonstrate that electron irradiation of graphene converts it to graphene oxide, an effect that is more pronounced at graphene‐liquid interfaces.^[^ ^124^ ^]^

Several radiation chemistry studies confirm this OH scavenging at the cost of graphene oxide formation.^[^ ^125^, ^126^, ^131^ ^]^ In turn, irradiation of graphene oxide appears to support radiolytic metal ion reduction.^[^ ^132^ ^]^ Hence, radical scavenging of graphene should not be read as an overall mitigation of radiolytic radicals: it may soften oxidative environments but does not mitigate reducing conditions. This correlates with the abundant studies of beam‐induced nucleation of noble metal nanostructures described in Section 7.2.

We finally note that graphene‐mediated scavenging is an interface effect. Consequently, it does not influence thick liquid layers, even when enclosed with graphene‐coated windows.^[^ ^129^ ^]^ It should also be borne in mind that graphene and similar LCs can exert high pressures on the sample,^[^ ^133^ ^]^ which may possibly alter the processes of interest.

#### 3.6.2 Liquid Flow: Can We Flush Away Radiation Chemistry?

Liquid flow has been considered a crucial experimental parameter for studying chemical reactions. The availability of advective solution exchange raised the idea of “flushing out” radiolysis products.

**Figure 9:**
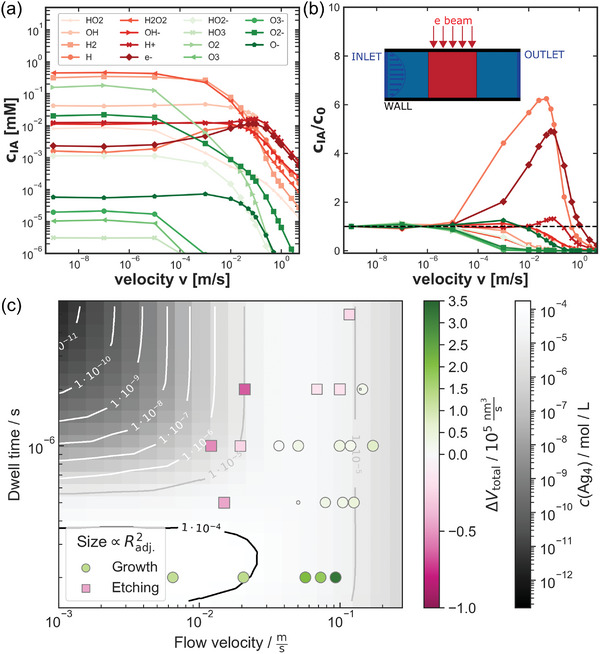
Liquid flow through the viewing window affects steady‐state concentrations in the irradiated area (*c*_IA_) as a function of the flow velocity. a) This is shown on absolute scale and b) relative to zero flow (c_0_) for water irradiated with 75 mGy s⁻^1^, revealing that stable products are reduced in concentration while reactive species are not. The inset in (b) shows a schematic cross‐section of an irradiated liquid channel with laminar flow. (a,b) reproduced from ref. [134], under a CC‐BY 4.0 license. This was verified for Ag nanostructure evolution, agreeing with kinetic modeling of Ag growth precursors (gray background). The flow impact is superimposed on the STEM dwell time effect discussed in Figure 7c. Reproduced after ref. [120].

Indeed, it has been shown that a liquid stream can affect the radiation chemistry of irradiated water as stable products like H_2_, O_2_, or H_2_O_2_ can be moved away from the viewing area. However, most reactive species such as solvated electrons, hydrogen‐, and hydroxide radicals are less affected (**Figure** **9**a). This is due to their extremely short build‐up period and the small mean distance these species can move before they encounter another species. Consequently, reactive species cannot be flushed away, and, under certain flow rates, the environment might become even more reactive (Figure 9b), as the self‐scavenging effect of these radicals that yield stable products is suppressed.^[^ ^134^ ^]^

Irradiation of nanosilver under AgNO_3_ flow supports this finding. Silver is a suitable material system, because its radiolytic growth depends on reduction by primary species, whereas etching is mediated by the secondary radiolysis product O_2._^[^ ^117^, ^119^ ^]^ Figure 9c demonstrates elevated growth with increasing flow velocity, suggesting flushing out of oxygen and accelerated precursor supply. This experiment also shows that STEM dwell time influences the growth rate, presumably because secondary products such as O_2_ require longer irradiation to build up. This is shown in Figure 7c and is supported by kinetic modeling of Ag_4_‐cluster concentration, a key species for silver growth.^[^ ^119^, ^120^ ^]^

If advection, as in micro‐ and nanochannel setups,^[^ ^135^, ^136^ ^]^ is unavailable, rapid diffusional materials exchange^[^ ^118^ ^]^ offers new avenues for influencing the radiation chemistry in a conventional bathtub LC design.^[^ ^137^ ^]^ While implications on radiation chemistry are still under study, we expect this again to affect long‐lived products that can travel large distances by diffusion.

Consequently, flow can be considered an important experimental parameter to tailor the chemistry within the observed volume by exploiting the different mean free paths and mobilities of chemical species generated upon electron irradiation. We can imagine, for instance, maintaining a constant supply of precursor solutions reacting with certain primary products^[^ ^134^ ^]^ and, thus, forming a defined reductive, oxidative, acidic, or alkaline regime.

## 4 Temperature Effects in LP‐TEM and LP‐SEM

Although radiolysis is the most obvious effect of irradiating a liquid in LP‐TEM, beam‐induced energy uptake can also cause unintended heating of the liquid. Furthermore, the deliberate control of temperature by heating and cooling the liquid is a common and useful LP‐TEM capability for quantifying materials phenomena, and interpreting these experiments involves understanding how radiation chemistry changes with temperature. These effects are discussed in the following sections.

### 4.1 Electron Beam‐Induced Heating

Electron irradiation is capable of causing temperature rises, potentially changing the rates of activated reactions dramatically due to the exponential Arrhenius dependence on temperature. Nonetheless, the possibility of electron beam‐induced heating is often overlooked in LP‐TEM literature.

**Figure 10:**
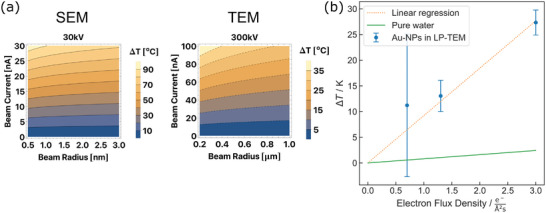
a) Simulation of beam‐induced heating of pure water at (left) 30 kV (SEM‐like) and (right) 300 kV (TEM‐like) conditions. Note the different axis and color bar scalings. Reproduced from ref. [71]. b) Measured electron beam‐induced heating in TEM (300 kV) of an ensemble of gold nanoparticles in a 140 nm thick liquid layer (blue dots), corresponding linear regression (orange, dashed line),^[^ ^63^ ^]^ and calculated beam‐induced heating of pure water under similar conditions.^[^ ^71^, ^141^ ^]^ Reproduced after ref. [63] and ref. [141].

Whether beam‐induced heating becomes significant and strongly depends on experimental conditions such as acceleration voltage, dose rate, as well as composition and thermal isolation of the sample. For ice under cryo‐TEM conditions, temperature rise during imaging is considered to be in the range of mK,^[^ ^138^ ^]^ unless the specimen surroundings exhibit poor heat conductivity or electron‐flux densities are high. In one such case, beam‐induced heating of ≈40 K at 120 kV at 50 $\frac{e^{-}}{{Å}^{2}s}$ was estimated to occur within milliseconds.^[^ ^66^ ^]^ On the other hand, **Figure** **10**a (right) shows minor beam‐induced heating of an aqueous liquid phase at 300 kV due to its high thermal conductivity and the relatively low inelastic scattering cross‐section.^[^ ^71^ ^]^ However, beam‐induced heating should not be ignored in general, because more often than not, LP‐TEM is used to study solid structures in liquid.

A first experimental indication of beam‐induced heating in LP‐TEM was reported by interpreting unexpected ZnO growth kinetics captured under high electron flux densities (10^5^ A m⁻^2^ $\approx 6.25\cdot 10^{3}\;\frac{e^{-}}{{Å}^{2}s}$ at 200 kV acceleration voltage).^[^ ^139^ ^]^ This indicates that high irradiation intensity alone may be sufficient to elevate temperature, even in a highly conductive bulk‐like liquid phase.

Electron beam‐induced heating in LP‐TEM was quantified at lower electron‐flux densities^[^ ^63^ ^]^ by employing charge‐mitigated parallel‐beam selected area electron diffraction (ED) on ensembles of gold nanoparticles.^[^ ^140^ ^]^ Diffraction was facilitated by thinning the liquid phase to 140 nm with an ambient air bubble. Such gas phases are often utilized to reduce the liquid layer thickness in commercial LCs to increase resolution. The results revealed significant electron‐beam‐induced heating that exceeds the expected magnitude of heating in pure water by an order of magnitude (Figure 10b).

This unexpected result suggests that, especially if the specimen of interest contains elements with high atomic numbers (*Z*), deliberate consideration of the balance between increased energy deposition and heat dissipation must be considered.^[^ ^3^ ^]^ The (approximately) tenfold increase in beam‐induced heating relates to the temperature determined at the gold nanocrystals themselves and an isolation effect caused by the micron‐sized bubble. Alternatively, secondary electron (SE) emission, discussed in Section 5, could provide substantial energy transfer to the surrounding liquid layer which may intensify beam‐induced heating near nanoparticles. Finally, micro‐ and nanoparticles can heat up faster than they cool down, which should be considered when regarding heat dissipation mechanisms.^[^ ^142^, ^143^ ^]^

When reducing the acceleration voltage, the inelastic scattering intensifies. This facilitates greater beam‐induced heating of pure liquid phases, e.g., during SEM as in the left panel of Figure 10a.^[^ ^71^ ^]^ Beam‐induced gold nanoparticle agglomeration in environmental SEM at 30 kV has been explained by local liquid evaporation caused by electron beam‐induced heating.^[^ ^144^ ^]^ We conclude that beam‐induced heating is a significant artifact in liquid‐phase SEM studies.

Overall, beam‐induced heating remains a poorly understood phenomenon in LP‐TEM that demands further clarification. Nonetheless, the status quo allows for a few implications. Beam‐induced heating is probably higher for heavier atomic number specimens – relevant to the numerous ensemble studies on high‐*Z* nanocrystals. Beam‐induced heating is most likely negligible, however, for experiments conducted in bulk liquid phases on low‐*Z* materials such as soft matter. We also note that the heat conductivity of the surrounding cell is crucial. This may favor LCs that enclose the specimen with a thick liquid layer or feature bulk material for heat dissipation. Such cell designs are typical for heating setups that allow for thermal control of reaction kinetics, and in such cells, beam‐induced heating is likely to be unimportant. Liquid flow may additionally provide a cooling effect to mitigate beam‐induced temperature rise. Still, on the other hand, large convection‐free gas bubbles could effectively isolate the system and enhance artificial heating.

### 4.2 Temperature‐Dependent Radiation Chemistry

**Figure 11:**
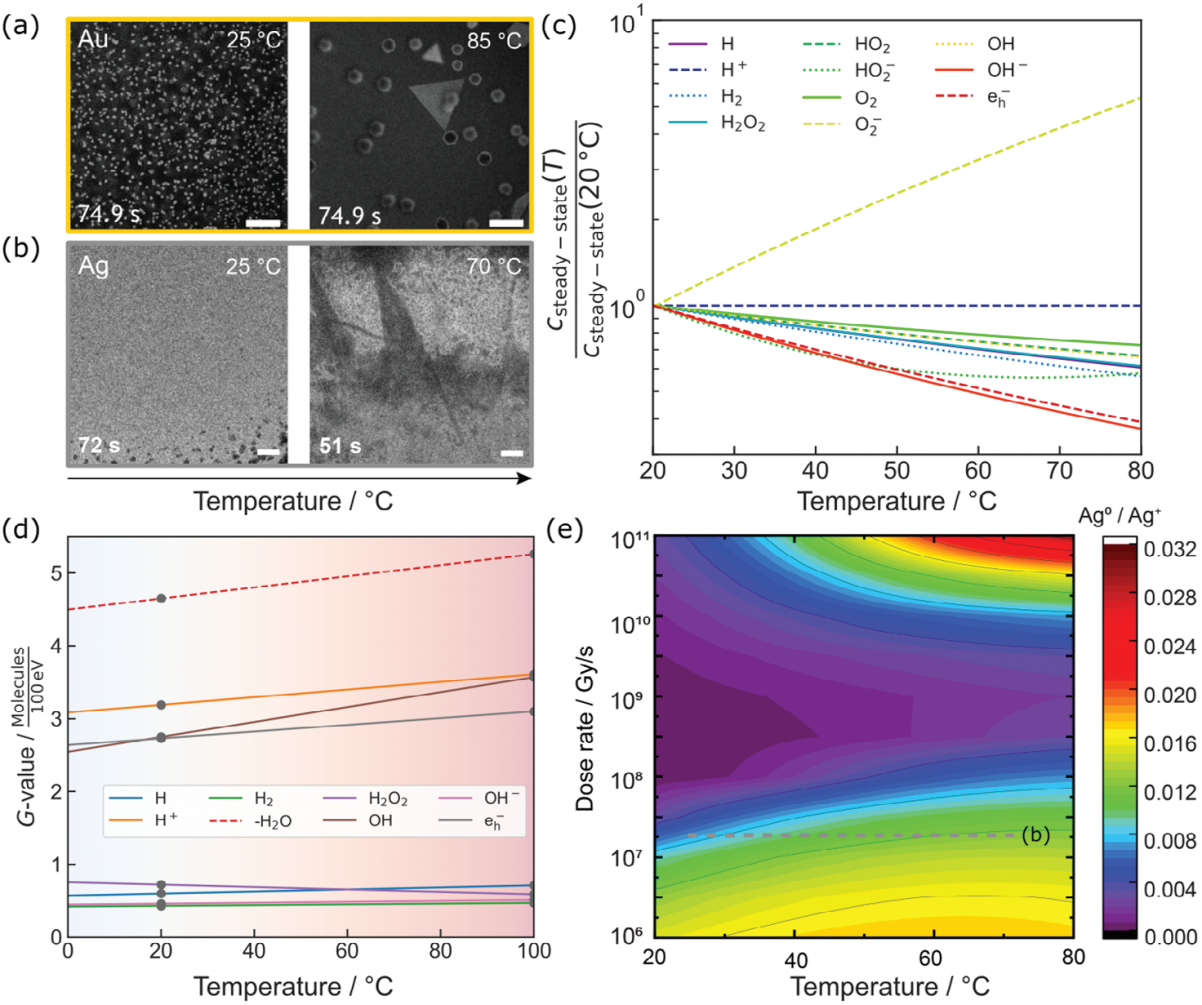
In situ LP‐TEM data showing beam‐induced growth of nanostructures at different temperatures, revealing changes in morphology. a) Au growth in an aqueous 1 mm HAuCl_4_ solution irradiated with 3.4 e^−^/(nm⁻^2^s). Used with permission of Wiley‐VCH, from ref. [145], copyright 2021 Wiley‐VHC; permission conveyed through Copyright Clearance Center, Inc. b) Ag growth in an aqueous 10 mm AgNO_3_ solution exposed to 2.8 · 10^7^ Gy s⁻^1^, reproduced with permission from ref. [146], copyright 2023, American Chemical Society. c) Relative changes of the steady state concentrations of radiolysis products in water with temperature (initial pH 1) at 5.6 · 10^7^ Gy s⁻^1^. Reproduced after ref. [148]. d) Temperature‐dependent *G*‐value evolution. The dotted values denote the *G*‐values obtained experimentally which are assumed to increase linearly with temperature, indicating more aggressive radiolysis at higher temperature. The water consumption is shown in absolute magnitude for comparison. After ref. [80]. e) Map of the ratio between the steady‐state concentration of Ag^0^ and Ag^+^ in 10 mm aqueous AgNO_3_ solution as a function of dose rate and temperature. The dashed line corresponds to the conditions shown in panel (b). Panel (e) is reproduced after ref. [146].

Besides beam‐induced heating, dedicated TEM holders and chips allow temperature control in LP‐TEM, opening up a powerful parameter space. Studies on metal nanoparticle evolution at elevated temperatures have demonstrated the temperature dependence of molecular diffusion, nanoparticle precursor concentration, and solubility, as well as faster surface reaction rates, leading to drastic morphology changes^[^ ^145^, ^146^ ^]^ (**Figure** **11**a,b). Figure 11a shows an image series of gold nanostructure formation from irradiated HAuCl_4_ solution at different temperatures, showing spherical Au nanoparticles growing at room temperature. In contrast, Au platelet formation is favored at elevated temperatures.^[^ ^145^ ^]^ Similar observations were made for Ag nanostructure formation in diluted AgNO_3_ solution. Here, hemispherical Ag particles grow at room temperature, whereas the growth mode changes to blade‐like dendrite formation at higher temperatures.^[^ ^146^ ^]^ To analyze the results of such temperature‐dependent LP‐TEM experiments, accurate modeling is paramount, as *G*‐values, diffusivity, and reaction rates change with temperature.

The first temperature‐dependent kinetic model incorporated temperature‐dependent *G*‐values, reaction rates, and H_2_ and O_2_ gas exchange with an adjacent bulk‐like gas bubble.^[^ ^80^ ^]^ These *G*‐values rely on linear extrapolation of experimentally determined values at 20 and 100 °C, which is justified by the behavior of Monte‐Carlo‐simulated *G*‐values.^[^ ^147^ ^]^ The underlying temperature‐dependent kinetic model for pure water only considers a limited number of reactants, yet, it was shown that the results were sufficient for LP‐TEM conditions.^[^ ^82^ ^]^ This is addressed in more detail in Section 6.2.

We show calculated temperature effects on radiolysis in Figure 11. As plotted in Figure 11c for water at a dose rate of 5.6 · 10^7^ $\frac{\mathrm{Gy}}{s}$ at an initial pH of 1, heating causes a decrease in all steady‐state concentrations except that of O_2_^−.[^ ^148^ ^]^ Similar trends are observed for a gold‐containing solution at pH 2.8.^[^ ^80^ ^]^ These findings must not be misunderstood to imply that irradiation causes less severe consequences at higher temperatures. Instead, all *G*‐values but H_2_O_2_ increase with temperature (Figure 11d),^[^ ^80^ ^]^ yielding higher water consumption and indicating more reactive conditions.

Subsequently, the model was adapted to describe temperature‐dependent silver radiation chemistry presented in Figure 11b, concluding that conditions are more reducing at elevated temperatures (Figure 11e).^[^ ^146^ ^]^ Moreover, a non‐linear effect of dose rate and temperature on the resulting steady state was shown, indicating that both effects must be considered simultaneously.

From these examples, we conclude that temperature‐dependent radiation chemistry simulations can help to interpret temperature‐controlled experiments. In contrast to heating, cooling is expected to yield opposing trends, favoring more stable radiolysis products and overall less radiolytic water splitting (Figure 11d). In addition, cooling decreases reaction rates. Although experimental studies are pending, especially in terms of linking with established cryo electron microscopy expertise, this suggests that cooling of water could be a way to mitigate radiolysis effects.

## 5 Secondary Electron Emission and Beam‐Induced Charging

Inelastic scattering in solid components such as cell membranes, electrodes, or nanoparticles influences LP‐TEM experiments by causing ionization that is accompanied by X‐ray emission, subsequent SE, and Auger electron emission. Charging effects due to SE emission from insulating materials such as silicon nitride used for liquid encapsulation can lead to the formation of an electric field.^[^ ^149^, ^150^, ^151^ ^]^ Similar to radiolysis, SE emission is not exclusive to electron irradiation but generally occurs under high‐energy irradiation.^[^ ^54^ ^]^

As a way to understand the effects of charging on imaging in electron microscopy, we note that charged membranes apply Lorentz forces on probing electrons. Consequently, they can be considered as artificial (small) electrostatic lenses.^[^ ^140^ ^]^ Changes in membrane charging cause a shift of the sharpest plane and blurring of the image. Thus, the electron microscope itself can be used to identify changes in membrane charging.^[^ ^140^, ^152^, ^153^, ^154^ ^]^

In terms of particle motion, the formation of an electric field will naturally trigger electrophoresis. This has the possibility of affecting particle motion inside^[^ ^155^, ^156^ ^]^ and outside^[^ ^157^ ^]^ the irradiated area. Beam current‐controlled slip‐stick particle motion (Lévy flights) at positively charged membranes is a prominent consequence.^[^ ^156^, ^158^, ^159^ ^]^ Charging of conductive nanomaterials is often estimated to be negligible,^[^ ^96^ ^]^ but ions in the solution could potentially change zeta potentials of nanostructures and thus cause slight net charging. Non‐aqueous systems may even exacerbate this phenomenon.^[^ ^98^ ^]^ In extreme cases, electron irradiation can cause peculiar effects like trapping of particles within the electron probe, referred to as electron‐beam tweezers.^[^ ^160^ ^]^

Emerging electric fields can similarly influence the motion of ions in the liquid phase^[^ ^161^, ^162^ ^]^ and cause ion redistribution alongside the field gradient.^[^ ^151^ ^]^ Moreover, charging changes the Gibbs free energy landscape, influencing observations like precipitation events.^[^ ^149^, ^150^ ^]^ The magnitude depends on the local field strength. However, as nonuniform electric fields cause drift of ions, nucleation and growth processes will be significantly different when observed in TEM, STEM, or ex situ.^[^ ^150^ ^]^ Depending on the diffusivity and lifetime of the respective species, accumulation of ions at large field gradients potentially may lead to spatially inhomogeneous reactions with radiolytic products.

In turn, charge dissipation and SE emission can trigger reactions adjacent to the irradiated area even if no aqueous diffusion paths are present, and especially when conductive membranes such as graphene are employed.^[^ ^83^, ^163^ ^]^ Conductive carbon layers are known to exhibit a bias‐dependent SE emission behavior.^[^ ^163^ ^]^ This should be borne in mind when applying graphene or derivatives as electrode material.^[^ ^164^ ^]^

We finally note that locally enhanced dose rates are expected at metallic nanostructures or electrodes due to SE emission, and these can potentially accelerate reactions that take place in close proximity to their interfaces.^[^ ^98^, ^151^, ^165^ ^]^ SEs may change the local dose rate to a small extent, thus having a minor effect on radiolysis.^[^ ^98^, ^165^ ^]^ In summary, the beam‐induced phenomena of SE emission and sample charging can affect LP‐TEM experiments in several ways, and the interpretation of data should be carried out keeping these various possibilities in mind.

## 6 Guidelines for Radiation Chemistry Simulations and Experimental Validation

To perform quantitative studies under irradiation, it is crucial to separate beam‐induced effects from phenomena that take place without the beam. Hence, in the following section, we will discuss how to assess the effects of radiation chemistry on the processes and structures under study. To do so, we first focus on setting up kinetic modeling. We then provide guidelines for kinetic model interpretation and finally discuss experimental approaches specifically for LP‐TEM. Insight from radiation chemistry modeling is crucial for meaningful outcomes, so we focus on this topic in depth and include methodological details. Readers mainly interested in interpreting simulation results and experimental verification of radiolysis are invited to jump to Section 6.4.

### 6.1 Representation of Radiation Chemistry by Differential Equations

To mathematically express the processes taking place during radiation chemistry, kinetic modeling has proven itself to be a feasible strategy. The change in concentration *c* of each chemical species *i* over time *t* is expressed by five main summands, representing formation and consumption via chemical reaction pathways, the generation of a species *i* by the interaction of the ionizing radiation, such as electrons, with liquid as sketched in Figure 2, diffusion, and liquid flow. Disregarding electric fields and assuming laminar flow, this can be stated as follows:

(11) $$\begin{array}{ccc} \frac{\partial c_{i}}{\partial t} & & =\sum_{j}k_{j}\;\left(\prod_{l}c_{l}\right)-\sum_{m\neq j}k_{m}\left(\prod_{n}c_{n}\right)+\rho \psi G_{i}+D_{i}\nabla^{2}c_{i}\\ & & +\nu \nabla c_{i} \end{array}$$

Here, *i* is the reactant of interest, *k* describes the rate constant of a specific reaction, *ρ* is the density of the aqueous phase, *D_i_* describes the diffusivity of *i*, and ν the flow velocity. ∇^2^ and ∇ are the (spatial) Laplacian and the gradient operators. The first two terms interconnect the concentrations of all reactants within the solution, spanning comprehensive reaction networks. In addition, the decay (second term) is naturally dependent on *c_i_* itself. The contribution of the electron beam requires knowledge of the *G*‐values. When applying the dilution approximation, as discussed in Section 3.3, those are zero for all but the primary species of the solvent.

For homogeneous systems with zero or negligible flow and diffusion, the last two terms become zero for all reactants. This leaves a core system of differential equations comprising three terms to be solved numerically when modeling radiation chemistry for LP‐TEM. The results of water for a homogeneous case (zero flow and diffusion) were shown in Figure 3a, whereas Figure 9a,b display an example comprising all terms of Equation (11). We note that the time dependence of each species concentration matched independent of the platform, within numerical tolerances of the solvers used, as shown for example in Figure 3a,b. We focus on this core system in the following.

### 6.2 Designing a Kinetic Model for Radiation Chemistry in LP‐TEM

When building a kinetic model for LP‐TEM, its scope must be clarified. In principle, two types of questions have been established that should be answered by a radiation chemistry simulation used for LP‐TEM. First, the simulation should be able to predict the radiation chemistry under irradiation. Second, modeling has proven beneficial in explaining observable phenomena. Although these may seem similar, the scientific workflow differs substantially.

For prediction, the model is used to map a parameter space that has not (yet) been accessed experimentally. Although experimental validation is often provided afterward (see below), the model does not have a priori knowledge of the result. This approach is, for instance, useful when planning experiments, as the microscopist can decide on suitable parameters based on the simulation outcome. Explanatory modeling, in turn, aims at understanding the experimental observations, and estimating the influence of the electron beam on the phenomenon. The distinction between prediction and explanation of course is blurred, and a successful model provides robust insights for both predicting and explaining experiments. Predictive models can only be valid within a certain parameter interval and thus should be tested experimentally, while explanatory models can also predict within their constraints. Hence, iterating between prediction and explanation is a powerful approach to yield robust and versatile models.

To achieve successful modeling outcomes, we recommend including as many groups of chemical species (clusters) as possible. The model should describe the interaction of these species with the primary species and capture their possible relaxation pathways, as was done in Section 3.6. Any complex reaction network can show a priori unexpected results, so including fast radical crosslinks between these clusters is important. Nonetheless, the model should remain as simple as possible to ensure simulation efficiency^[^ ^82^ ^]^ and to prevent false conclusions that may arise from interpreting unnecessarily complicated data,^[^ ^166^ ^]^ hence supporting good scientific practice.^[^ ^167^ ^]^ In LP‐TEM simulations, this mainly refers to the number of reactants, which defines the parameter space of the model (see Equation 11). However, it is not trivial to identify a sweet spot between sufficient detail and too much simplicity. Experimental benchmarking and model sparsing^[^ ^82^ ^]^ are possible approaches to solve this problem.

**Figure 12:**
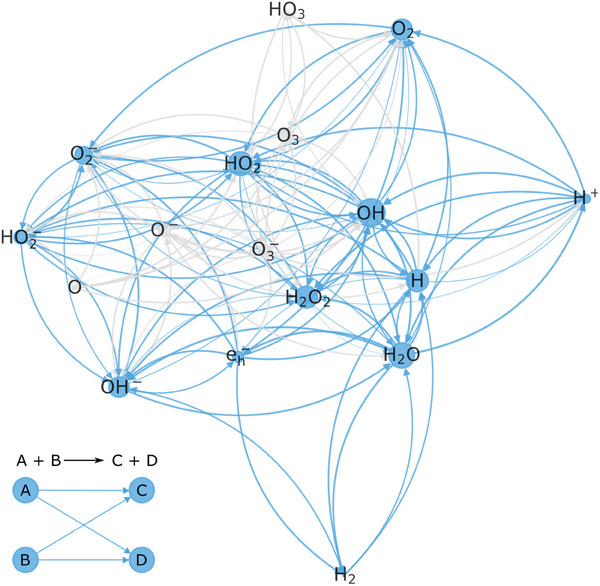
Sparsing a kinetic model for LP‐TEM related radiation chemistry illustrated by a graph representation of a reaction network for pure water. The chemicals and related reaction pathways found irrelevant for LP‐TEM are shown in gray, and relevant species and pathways are shown in blue. The edge thickness reflects the log‐scaled reaction rate constant. The node size corresponds to the betweenness centrality. Reproduced (with color changes) from ref. [82], CC‐BY 4.0 licensed.

Sparsing the pure water model revealed that LP‐TEM conditions are accurately pictured by including only 12 out of the 16^[^ ^78^ ^]^ or 17^[^ ^83^ ^]^ reactants usually considered. As illustrated in a graph representation of the reaction network (**Figure** **12**), the relevant species apart from the solvent and the primary products (see Equation 4) are only secondary products (HO_2_^−^, O_2_, and O_2_^−^). This is the same group used to form the temperature‐dependent aqueous kinetic model of water discussed in Section 4.2. However, accurate modeling of steady‐state decay requires incorporating additional reaction pathways, e.g., by adding the para‐oxygen radical.^[^ ^82^ ^]^

Likewise, it may be beneficial to constrain the reactions to relevant interactions. Investigating the contribution of single chemical reactions is possible by analyzing the simulation outcome to calculate the materials throughput of individual reactions.^[^ ^119^ ^]^ This could, in principle, be used to minimize the complexity of a reaction set further or aid in identifying redundant species.

### 6.3 Practical Implications for Radiation Chemistry Modeling

When building or extending a kinetic model, it is feasible to account for a few general design guidelines that can simplify the workflow. First and foremost, the conservation of mass and charges must be considered. This relates to both the chosen set of *G*‐values and all reactions included. Atom/mass balance for *G*‐values should be checked separately for each sort of atom in the solvent. If the sum formula of the solvent contains *n* atoms of an element and irradiation creates *m* primary products which contain the element *j* times, this can be done via:

(12) $$G_{\mathrm{solvent}}+\frac{1}{n}\;\left(\sum_{i}^{m}j_{i}G_{i}\right)=0$$

To fulfill this equation, the *G*‐value of the solvent (*G*_solvent_) must be negative, which can be pictured as a consumption value. We show as an example this calculation for the *G*‐values of water listed in Table 1. In the calculation, e_h_^−^, H^+^, and OH^−^ are regarded without a hydration shell:^[^ ^78^ ^]^

(13) $$\begin{array}{ccc} H\;\mathrm{balance}:\;\;\;G_{H_{2}O} & & +\frac{1}{2}\;\left(G_{H^{+}}+G_{OH^{-}}+G_{H}+G_{\mathrm{OH}}+2G_{H_{2}}\right.\\ & & \left.+2G_{H_{2}O_{2}}+G_{HO_{2}}\right)=0\;\; \end{array}$$

(14) $$\begin{array}{ccc} O\;\mathrm{balance}:\;\;\;G_{H_{2}O} & & +1\;\left(G_{OH^{-}}+G_{\mathrm{OH}}+2G_{H_{2}O_{2}}+2G_{HO_{2}}\right)\\ & & =0\;\; \end{array}$$

While the dilution approximation holds, *G*‐values that only consider the solvent are sufficient. For non‐diluted solutions, *G*‐values become composition‐dependent and may therefore change during irradiation.

Checking the simulation outcome for violations of physical constraints such as mass and charge balance is a powerful way to reveal possible errors within the model. Disregarding these boundary conditions can quickly lead to unphysical simulations without predictive power. Hence, the model description should state and justify any purposely introduced violations. Similarly, double‐checking against violation of other boundary conditions (e.g., concentrations and dose rates for which the dilution approximation is/remains valid) is advised. Again, any conditions and constraints should be well thought out and openly stated alongside the simulation outcome.

An overview of typical platforms used for LP‐TEM‐related radiation chemistry simulations is given in **Table** **2**. The software solutions listed differ in key aspects. One is source code accessibility and professional support (open access). Another is the necessity or possibility of coding (a coding interface vs a graphical user interface). This becomes relevant when an extension of the software capabilities is desired, for instance to validate the simulation constraints. Certain tools provide functionality to simplify the hunt for typing errors that could violate boundary constraints^[^ ^83^ ^]^ or to verify that charge‐ and mass conservation holds (typo‐, charge‐, and mass‐balance checking). It is important to note that all tools can perform simple, 0D (isotropic) simulations, and the solution obtained for Equation (11) should not vary for identical simulation conditions and solver accuracies, as shown for AuRaCh and MATLAB in Figure 3a,b. This enables the user to make a decision between software solutions based on individual preferences. However, if multidimensional modeling is needed, for instance, to account for geometries, inhomogeneous irradiation, effects outside the irradiated volume, or spatial motion, this narrows down the number of available software solutions.

**Table 2:** Platforms used for radiation chemistry modeling. * indicates free‐of‐charge access possible.^[^ ^168^ ^]^

Platform	Open access	Coding interface	Graphical user interface	Typo‐, charge‐, and mass‐balance checking	Multi‐dimensional modeling	Exemplary usage in LP‐TEM
Wolfram Mathematica	no	yes (own language)	no	no	no	refs. [78, 96, 117, 146]
MathWorks MATLAB	no	yes (own language)	no	no	no	refs. [78, 80, 81, 97, 99, 123, 169]
COMSOL	no	yes (MATLAB‐compatible)	partly	no	yes	refs. [78, 106, 107, 134, 149]
AuRaCh	yes^[^ ^83^ ^]^	yes (Python)	yes	yes	no	refs. [70, 82, 83, 88, 119]
CHEMSIMUL	no*	no	yes	yes	no	refs. [90, 170]
MCPA Software Ltd FACSIMILE	no	yes (own language)	yes	no	yes	n.A.

To yield a steady‐state formation, each chemical must gain access to at least one formation and one decay route;^[^ ^83^ ^]^ Otherwise, a reactant will be consumed completely, or a product will eventually accumulate all matter. In such cases, the reaction route is set as a constraint. While it is still possible to gain insights into the time domain of such models (kinetics), their thermodynamic interpretability (concentrations) is strongly diminished.

To build a suitable kinetic model, reliable kinetic information is crucial. This is a limitation for comprehensive model building, as parameters may not be available for many interactions. However, if the related reactant concentrations remain extremely low throughout the desired scenario, the reaction could be neglected due to minor importance. Differences in chemical potential between reactants and products can also help in assessing the importance of reaction pathways.^[^ ^80^, ^123^ ^]^ However, such compromises can cause pitfalls of unexpected network pivots and should be considered a stopgap solution.

Rate constant data based on pulse‐radiolysis studies is widely available within publications of the radiation chemistry community. A comprehensive summary of older literature data is available in the NIST solution kinetics database.^[^ ^171^ ^]^ Moreover, many typical solvents and solute combinations have already been as kinetic models for LP‐TEM. A summary is provided in **Table** **3**.

**Table 3:** Solvent/cluster combinations realized in kinetic models for radiation chemistry in LP‐TEM. ROH, TRIS, BIS‐TRIS, PEG.SH, MeOH, DMF, and IPA abbreviate alkanoles, tris(hydroxymethyl)aminomethane, Bis‐(2‐hydroxy‐ethyl)‐amino‐tris(hydroxymethyl)‐methane, polyethylene glycol thiol, methanol, dimethylformamide, and isopropanol.

H_2_O^[^ ^78^, ^80^, ^82^ ^]^+	Cl^−[^ ^70^, ^82^, ^96^, ^97^, ^172^ ^]^
	Br^−[^ ^70^, ^97^ ^]^
	I^−[^ ^97^ ^]^
	NO_3_^−[^ ^70^, ^146^ ^]^
	SO_4_^2‐[^ ^169^, ^173^ ^]^
	Fe^2+/3+[^ ^88^, ^90^, ^174^ ^]^
	Na^+^ / Cs^+[^ ^70^ ^]^
	Ce^3+/4+[^ ^89^, ^99^ ^]^
	Au^[^ ^80^, ^83^, ^98^ ^]^
	Ag^[^ ^117^, ^146^ ^]^
	ROH^[^ ^98^, ^117^, ^119^ ^]^
	TRIS^[^ ^173^ ^]^
	BIS‐TRIS^[^ ^90^ ^]^
	PEG‐SH^[^ ^175^ ^]^
	AuCl_4_^−[^ ^82^, ^83^ ^]^
	AgNO_3_^[^ ^119^, ^146^ ^]^
	FeCl^+[^ ^174^ ^]^
MeOH^[^ ^106^ ^]^	
DMF^[^ ^106^ ^]^	
IPA^[^ ^107^ ^]^	

Naturally, measured rate constants imply a distinct uncertainty. Hence, it is worth performing a sensitivity analysis of those on the outcome of simulations. Such an analysis for H_2_SO_4_‐containing solutions shows that a tenfold variation is irrelevant for H_2_ and H_2_O_2_ steady‐state concentrations.^[^ ^169^ ^]^ This suggests that agreement on an order of magnitude is important for these particular rate constants.

### 6.4 Interpretation of Kinetic Models

Depending on the model parameters, temporal evolution may be trivial when there is rapid steady‐state formation. However, this does not imply that steady‐state formation occurs immediately under all conditions, even at high dose rates. In particular, if slow processes take place in the sample (such as long‐term degradation of soft matter)^[^ ^98^ ^]^ or diffusion‐controlled steady‐state formation outside the electron beam,^[^ ^78^, ^176^ ^]^ the time‐dependence can be more difficult to determine. In such cases, both dose rate and absolute dose become important and should be considered, similarly to cryo TEM.^[^ ^66^ ^]^

In principle, the difference between dose rate‐dependent steady states and their transients can be interpreted analogously to the difference in reaction kinetics (time domain) and shifts in the Gibbs free energy (energy domain). These transients describe changes under continuous exposure with a given dose rate. Thus, the concentrations at a given time point in the transient can be expressed as a function of the accumulated dose during the time interval the sample was irradiated. As soon as forward and backward reactions balance, the net concentrations remain constant (steady state), and the radiation chemistry becomes independent of time. This is the typical scenario of a dynamic equilibrium. Thermodynamically, dynamic equilibria describe local energy minima within the Gibbs free energy landscape. As irradiation changes the steady state with the dose rate, this can be interpreted as a shift of this free energy minimum by irradiation.^[^ ^83^ ^]^

When interpreting steady‐state concentrations, it is important to remember that this dynamic equilibrium describes balanced concentrations after the system relaxes under consideration of all the available reaction pathways. Hence, we must never confuse steady‐state concentrations with the reactivity or the apparent relevance of a reactant in the system. This can be pictured by regarding e_h_^−^, arguably the strongest reductant available. Being a primary species, it is constantly generated, but naturally, its concentration decays rapidly due to its extremely high reactivity. As a consequence, its steady‐state concentration remains low, as in examples such as in Figure 3a,b.

Graph theory offers a mathematical approach to accessing the true relevance of a reactant within a reaction network. A network is represented by nodes connected via edges, where nodes represent reactants and edges represent reactions. By analyzing the shortest path between different nodes along these edges, a measure of the centrality of each node can be obtained. This allows for estimating the involvement of a reaction in the complex interplay of the network.^[^ ^83^ ^]^ Figure 12 illustrates this for the reaction network of pure water. Here, the node size is defined by the betweenness centrality of each reactant,^[^ ^177^ ^]^ which accounts for reaction‐rate constants on a log scale. It is clear from the figure that e_h_^−^ obtains a higher centrality than H_2_, a main product in water radiolysis. This analysis treats the reaction network irrespective of initial concentrations or dose rate, but nonetheless, can be used to visualize time‐dependent concentration evolution using adjusted node size definitions.^[^ ^119^ ^]^

If a tailored model is unavailable, extrapolating steady‐state conditions to an adjacent liquid system is tempting. This is particularly common with simulations describing plain water. However, irradiation exposes systems to the primary species formed, in contrast to a beaker system where chemicals can be subsequently exposed to an equilibrated state. Hence, the nature of the steady state of the system strongly depends on the magnitude of alternative reaction pathways that a diverging chemical environment can enable. In particular, if the specimen reacts rapidly with primary species, this can trigger a (potentially involuntary) scavenging of reactive chemicals (see Section 3.6); if such scavenging is negligible, extrapolations of steady‐state results are feasible.

### 6.5 Experimental Assessment of Electron Beam Effects

We now discuss the experimental validation of electron beam‐driven effects (and thus their models) in LP‐TEM. This is the first stage of correctly understanding beam effects, and achieving a correlation of simulations with experimental results requires accurately measuring the dose rate used in the experiment. The dose rate can be converted from the electron‐flux density measured from the microscope interface in the absence of a sample as described in Section 2. For radiation chemistry networks, it is then essential to identify indicator quantities such as species concentrations or ratios that are experimentally accessible.

**Figure 13:**
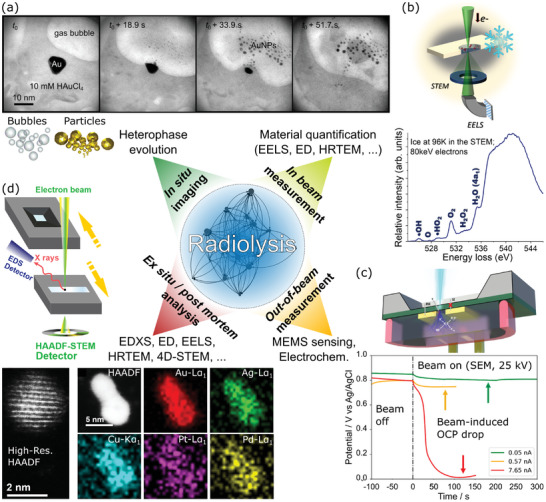
Overview of different approaches to experimentally accessing radiolysis products with illustrative examples. a) Evolution of radiolytic phase transitions is illustrated on dynamics at a solid‐liquid‐gas interphase. Reproduced from ref. [94], with permission from A. Hutzler, CC BY‐NC 4.0 licensed. b) In‐beam measurement of radiolysis products is demonstrated during cryo electron microscopy. Reproduced with permission from ref. [138], copyright 2023, American Chemical Society c) MEMS‐based out‐of‐beam sensing of radiolysis products, here highlighted on an electrochemical measurement during SEM. Reproduced with permission from ref. [169], copyright 2023, American Chemical Society. d) ex situ / *post mortem* materials characterization by investigation of structures on the chip membranes after the experiment, often by disassembling the LCs, and shown here for HR‐STEM and EDXS of high‐entropy alloys. Used with permission of The Royal Society of Chemistry from ref. [178], permission conveyed through Copyright Clearance Center, Inc. Copyright 2023, The Royal Society of Chemistry.

The beam‐induced steady state decays rapidly outside the electron beam, but capturing effects within the irradiated volume is challenging. Several approaches are summarized in **Figure** **13**, classified as in situ imaging, ex situ analysis, and in situ measurements. The latter is divided into measurements performed within the electron beam (in‐beam analysis) and those that measure only partially or close to the irradiated volume (out‐of‐beam measurements).

The most prominently used strategy to investigate beam effects is to observe heterophase evolution, usually referring to bubble or nanoparticle formation,^[^ ^71^, ^78^ ^]^ or their interplay. Figure 13a shows an image series of a gold platelet being etched by a gas phase in aqueous 1 mm HAuCl_4_ solution.^[^ ^94^ ^]^ This phenomenon could not be explained by radiolysis simulations of pure water that indicated only H_2_ and O_2_ formation: including Au and Cl clusters in the model revealed the formation of hydrochloride as well as strong oxidants such as OH radicals that led to complexation of oxidized gold ions.^[^ ^83^ ^]^ Such indicator reactions yield information about the environment within the irradiated volume. Similar to classical chemistry, precipitation can highlight the presence of distinct reactants. For instance, this approach was used to deduce an elevated presence of OH^−^ ions under irradiation^[^ ^89^ ^]^ and to classify the dose rate‐dependent interplay of radiolytic acidity and redox environment.^[^ ^88^ ^]^ Yet, this requires a precise understanding of these reactions in the first place. Drawing quantitative conclusions about such phenomena is challenging, and the chemical composition of these heterophases is not always clear. Hence, in‐beam measurements such as electron energy loss spectroscopy (EELS)^[^ ^91^, ^99^, ^138^ ^]^ or crystal structure analysis via ED^[^ ^63^, ^179^ ^]^ and HRTEM^[^ ^83^, ^117^, ^180^, ^181^ ^]^ facilitate direct verification of beam‐induced processes (Figure 13b).

MEMS‐based sensing (Figure 13c) offers another approach to gaining information on the chemical environment formed by radiolysis and enhances the number of accessible measurement techniques. One outcome of such a measurement is a demonstration that beam‐induced heating in bulk liquid layers does not heat up the entire LC architecture,^[^ ^182^ ^]^ suggesting that beam‐induced heating is a local phenomenon that must be measured in‐beam. Electrochemical approaches are also compelling: a specially designed SEM LC enabled quantification of H_2_ generation even in a bulk solution, and measurement of a beam‐induced change in open‐circuit potential.^[^ ^169^ ^]^ In general, it is challenging to restrict the measured signal to the irradiated volume, yet, in combination with appropriate modeling, these techniques help the researcher reconstruct the conditions under beam exposure.

Ex situ approaches enable *post‐mortem* sample characterization. Figure 13d shows an example of a post‐synthesis spectrum image series observed in LP‐TEM of high entropy alloy (Au, Ag, Cu, Pt, Pd) nanoparticles grown from a precursor‐ligand solution via beam‐induced reduction, yielding particles like those obtained by NaBH_4_ reduction in flask‐chemistry.^[^ ^178^ ^]^ As the sample is usually dried at this point, measurements can be made using conventional electron microscopy that can allow for higher resolution and signal integration times. This simplifies, for instance, the acquisition of high‐resolution data and EDX spectra^[^ ^108^, ^178^, ^180^ ^]^ or 4D‐STEM.^[^ ^109^ ^]^ However, depending on the LC architecture, additional sample transfer may become relevant to allow safe exposure of the ex situ sample to the electron microscope. This is because simple removal of a single membrane in a commercial LC holder featuring a chip sandwich structure and liquid channels is generally not advised without additional safety precautions. ex situ sample investigations are of course not limited to TEM, so additional post mortem characterization is possible, for instance by optical techniques or scanning probes.^[^ ^183^ ^]^ This renders *post‐mortem* analysis a powerful approach to complement LP‐TEM studies beyond its use for assessment of beam‐induced artifacts.

## 7 Consequences and Implications for Quantitative Liquid‐Phase Electron Microscopy

**Figure 14:**
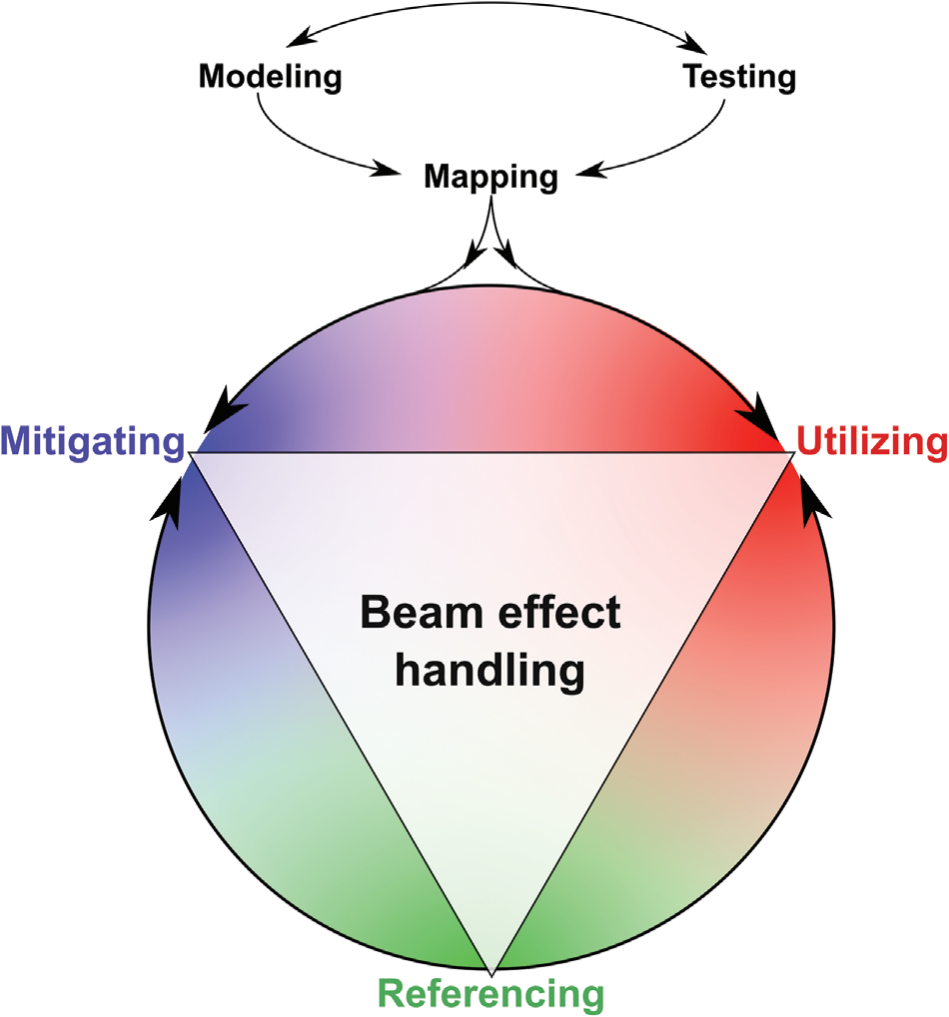
Schematic of the proposed workflow to handle beam effects: beam effect mapping and external referencing are suggested irrespective of the chosen beam handling strategy (mitigation or utilization).

As beam effects have become more recognized in LP‐TEM, two main strategies have emerged to handle these effects constructively. Many experiments involve substantial effort to mitigate beam effects, while others utilize beam effects to study phenomena. Both strategies are important in advancing scientific knowledge; in either case, careful assessment of the beam influence itself is mandatory. We discuss these approaches with reference to the schematic depicted in **Figure** **14**.

### 7.1 Mitigation of Electron‐Beam Induced Effects

To minimize the influence of the electron beam, it is intuitive that the electron flux experienced by the sample should be reduced. However, lowering the total dose is also important in cases where changes to the sample accumulate with the total dose. Such cases could include systems in which radiolysis species diffuse far from the irradiated area, or in which the sample or membranes are damaged by irradiation.

Minimizing the dose rate can be achieved using a lower flux and a more sensitive detector. Increasing the dark time between frame acquisition^[^ ^184^ ^]^ reduces the average dose rate, although the irradiation while acquiring each frame or pixel value in STEM can still trigger substantial radiolysis. Dose‐efficient scanning techniques such as sub‐sampling approaches^[^ ^81^, ^185^ ^]^ or event‐responsive dwell time adjustment^[^ ^186^ ^]^ similarly reduce the average dose rate and total dose to the sample, although the areas sampled still experience high dose rates.

A key question is whether a dose rate can be low enough to avoid chemical or other changes, yet high enough to acquire usable data. As the dose rate decreases to values well below what is typically used for imaging, say below 1 $\frac{\mathrm{Gy}}{s}$, the simulations we have presented show that changes in chemistry are still expected ‐ although naturally with weaker effects compared to higher dose rate values. Radiolytic acidity illustrates this point, since (as discussed in Section 3.2) a dose rate below ≈10^3^ $\frac{\mathrm{Gy}}{s}$ is required to yield a steady state where the acidity would be governed by aqueous autoprotolysis. At 300 kV, this translates to ≈10^−4^ e^−^ Å⁻^2^s^−1^ (see Figure 1). Other beam effects, such as beam‐induced heating, can also decrease to irrelevance at a low dose rate, although charging and radiolysis may be harder to bypass. Under certain experimental conditions, it may therefore be sufficiently accurate to treat beam effects as insignificant allowing the study of strong chemical effects without electron beam influence.^[^ ^187^ ^]^

In designing such experiments, modeling helps to suggest the conditions that are optimal, with respect to beam effects, for the specific phenomenon under study. Modeling may also help to improve the efficiency of experimentation. Systematic variation of experimental parameters is critical to mapping out the system response and drawing meaningful conclusions, regardless of whether the chosen beam effect strategy is mitigation or exploitation. However the parameter space may be broad and is also highly specific to the experimental design, so modeling can inform the choice of parameters to explore. This has, for instance, been successfully implemented for critical radius measurements of metal nanoparticles,^[^ ^83^ ^]^ temperature‐dependent polymer assembly,^[^ ^107^ ^]^ emulsification,^[^ ^188^ ^]^ or electrochemically‐driven nanofiber self‐assembly.^[^ ^183^ ^]^

Membrane charging can be mitigated by employing conductive membranes or a conductive membrane coating.^[^ ^140^ ^]^ However, this may cause charge dissipation and trigger redox events adjacent to the irradiated volume.^[^ ^83^ ^]^ This should be considered when referencing structural changes against a different region within the same LC or graphene LC grid. Moreover, increasing the distance between the sample and the membrane reduces the magnitude of the membrane‐originated charge field.^[^ ^150^ ^]^ This can be achieved, in principle, by employing thick layers of viscous solutions,^[^ ^182^ ^]^ but this may not be applicable to many experiments and is accompanied by further resolution loss.

The optimal conditions for data collection are clearly highly dependent on the specific phenomenon under study, so we refrain from giving specifics. However, physical considerations can support choosing the most dose‐effective imaging mode.^[^ ^189^ ^]^ A general objective, if feasible, is to use low‐*Z* instead of high‐*Z* “support” structures (electrodes, markers, etc.) to improve contrast, thereby reducing the dose, and mitigating SE emission and thus radiolysis and heating.

### 7.2 Utilizing the Electron Beam

Many informative experiments harness the inevitable beam effects to trigger desired phenomena. In combination with tailored radiation chemistry knowledge, the beam can generate distinct conditions or trigger effects of interest, even substituting radiolysis products for the reactants used in benchtop experiments.

**Figure 15:**
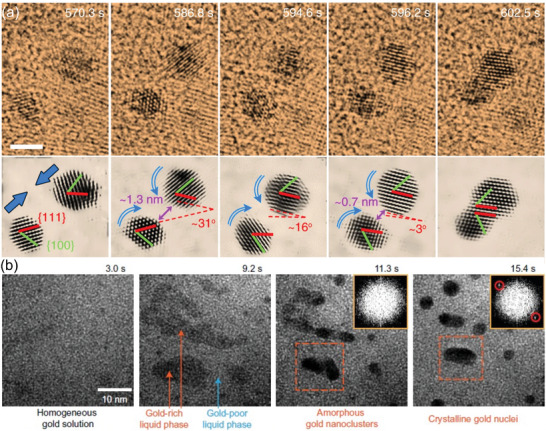
a) Oriented attachment of gold nanoparticles at a {111} surface in an HAuCl_4_ solution, where irradiation yields Au^0^ precursors. The particles first approach, rotate during pre‐alignment and jump to contact evolving into a twin structure (scale bar: 2 nm). Reproduced from ref. [191], under a CC‐BY 4.0 license. b) Beam‐induced, non‐classical multistep nucleation of gold nanoparticles in a supersaturated Au^0^ solution. Nanoclusters emerge from metal‐rich phases, which then crystallize. Insets show Fourier analyses of orange boxes, indicating subsequent crystallization. Reproduced from ref. [192], with permission from Springer Nature Customer Service Center. Copyright 2017, Springer Nature.

**Figure 16:**
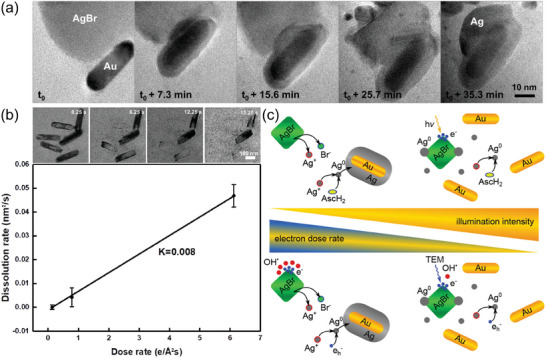
a) Use of the electron beam as an alternative stimulus to trigger a chemical Au@Ag core‐shell synthesis using CTAB‐stabilized gold nanorods in an irradiated AgNO_3_ environment. Combining Ag^+^ and Br^−^ leads to AgBr precipitation prior to irradiation. Reproduced with permission from ref. [180], CC‐BY‐NC‐ND 4.0 licensed by the original authors. b) Linear dependency of the electron flux density on the akaganeite mineral dissolution rate. Reproduced with permission from ref. [90], CC‐BY‐NC‐ND 4.0 licensed by the original authors. c) Schematics of electron beam irradiation of CTAB‐containing AgNO_3_ solutions, either enabling Au@Ag core‐shell synthesis (shown in (a)) or Ag platelet growth, compared with analogous UV‐photochemical processes. Reproduced with permission from ref. [180], CC‐BY‐NC‐ND 4.0 licensed by the original authors.

The most familiar example of this is the use of electron beam‐induced solvated electrons and H‐radicals as reductants for particle nucleation studies, leading, for instance, to mechanistic insights into particle growth and assembly. Combining this well‐established application of radiation chemistry^[^ ^73^ ^]^ with LP‐TEM has led to significant insights into nanoparticle evolution. Utilizing the electron beam often allows for high electron flux densities, enabling high‐resolution imaging. Prominent results include understanding the details of oriented attachment,^[^ ^190^, ^191^ ^]^ where observations reveal that particles first orient and then merge along aligned facets (**Figure** **15**a); study of non‐classical nucleation phenomena,^[^ ^103^, ^119^, ^192^, ^193^, ^194^, ^195^ ^]^ showing nanoparticle emergence via multistep processes that involve amorphous and crystalline intermediates (Figure 15b);; and growth processes for core‐shell nanostructures,^[^ ^180^, ^196^, ^197^, ^198^ ^]^ illustrated in **Figure** **16**a. Similarly, radicals can be exploited in polymerization studies, as described in Section 3.4.

Other types of material reactions can also be driven by beam‐induced radiation chemistry, with outcomes such as facilitating accelerated stress tests of materials during corrosion.^[^ ^199^ ^]^ Controlled beam‐induced corrosion can even be used for micropatterning surfaces.^[^ ^200^ ^]^ Beam‐induced H_2_ has been harnessed to study nanocatalysts in situ.^[^ ^181^ ^]^ It is well known that the etching induced by radiation chemistry depends on the electron‐flux density,^[^ ^90^, ^179^, ^201^, ^202^, ^203^ ^]^ as shown in Figure 16b for beam‐induced akaganeite mineral dissolution. Such experiments benefit greatly from radiation chemistry modeling in obtaining a quantitative and mechanistic understanding of the processes involved.

As a final example, OH radicals have been used successfully to mimic radical scavenger‐driven reaction pathways in photochemistry (Figure 16c). Here, electron irradiation of an aqueous silver nitrate solution containing cetrimonium bromide (CTAB)‐stabilized Au nanorods could mimic results for Ag shell growth obtained on the benchtop using a reducing agent. Upon mixing AgNO_3_ with a CTAB‐containing solution, AgBr particles precipitate as intermediates. Unlike exposure to light, electron irradiation does not induce a direct reduction of AgBr to silver particles. This is because OH radicals act as scavengers of electrons excited into the conduction band of AgBr. Subsequently, H radicals and solvated electrons reduce solvated silver ions, removing the necessity for additional reductants. The process yielded Au‐Ag gold‐silver core‐shell particles as expected from ex situ UV–vis studies.^[^ ^180^ ^]^ As illustrated here, such mechanistic understanding requires detailed knowledge of the radiation chemistry environment facilitated by a systematic combination of reaction parameter sweeps and kinetic modeling.

### 7.3 The Gold Standard: Verification Experiments

**Figure 17:**
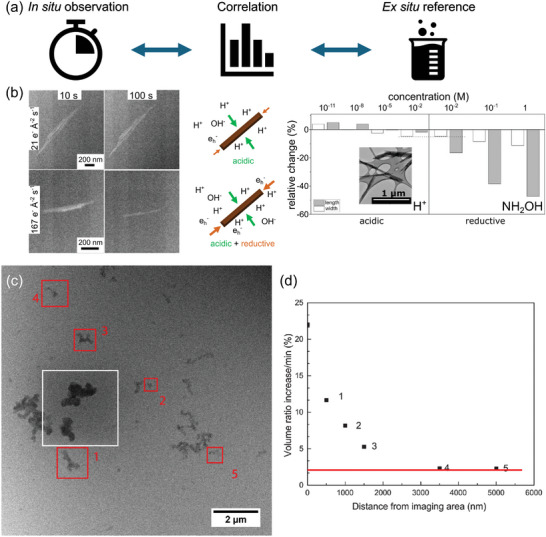
a) Schematic of an ex situ verification experiment for LP‐TEM data. b) Example of goethite degradation investigated via the schematic in (a). Left: in situ results under different electron flux densities, matching pH and reductive environment changes (center) obtained ex situ (right). Reproduced from ref. [88], CC‐BY 4.0 licensed. c) ex situ analysis of CoCO_3_ particle nucleation. The white rectangle denotes the area irradiated during the experiment. By analyzing the relative volumetric growth in different areas adjacent to the irradiated volume (red marks), growth rates unaffected by electron beam interactions are obtained as a function of distance to the irradiated area (d). Panel (c) and (d) are reproduced with permission from ref. [204], CC‐BY‐NC‐ND licensed by the original authors.

The most convincing verification that in situ observations are meaningful is a comparison experiment (**Figure** **17**a). This should be performed regardless of the chosen beam effect strategy. Ideally, it is done in a system thermodynamically isolated from the LC to avoid cross‐talk due to dissipation effects (see Section 7.1) or diffusion of long‐lived reaction products.^[^ ^176^ ^]^ This naturally requires a statistically relevant number of observations. For instance, oxidative bubble etching of gold nanorods in the presence of HBr was supported by collecting ex situ particle statistics.^[^ ^95^ ^]^ Similarly, radiolysis‐driven goethite mineral degradation was quantified through systematic ex situ benchmarking of different environments varying in pH and reductant concentration (Figure 17b).^[^ ^88^ ^]^ Oxidative stress was mimicked by UV‐mediated H_2_O_2_ fission, yielding OH radicals.^[^ ^129^ ^]^

While such benchmarking is straightforward for comparison of ex situ reaction products, dynamics are obviously more difficult to reference. Possible strategies include correlative microscopy^[^ ^205^ ^]^ or identical location TEM studies.^[^ ^206^, ^207^ ^]^ Given the vast experience in soft matter and biology with cryo‐TEM, its combination with LP‐TEM provides powerful insights.^[^ ^208^ ^]^ Cryo‐ and identical location TEM offers only snapshot information and thus can only verify dynamics within a limited temporal resolution, which LP‐TEM complements. In the case of correlative in situ microscopy, to exclude radiation artifacts on dynamics, it should be borne in mind that complementary techniques must not harness ionizing radiation, such as γ‐, α‐, X‐ray‐, or proton beams. Even deep UV light triggers radiolysis because ionization energies for water are lower than 10 eV.^[^ ^209^ ^]^ Correlations with light microscopy,^[^ ^210^ ^]^ dynamic light scattering,^[^ ^208^ ^]^ and mass spectrometry^[^ ^178^ ^]^ have been successfully applied.

A further powerful strategy to assess beam effects is to compare the experimental outcome within the LC between irradiated and non‐irradiated regions after the experiment has concluded. As shown in Figure 17c, for a study of CoCO_3_ nanoparticle formation, systematic particle size analysis as a function of distance to the irradiated area allows a back‐calculation to zero Gy (Figure 17d).^[^ ^204^ ^]^ The measurement aimed to determine the distance beyond which the electron beam did not affect the particle formation process. This was found to be beyond ≈3 µm from the irradiated area,^[^ ^204^ ^]^ consistent with 1D diffusion analyses of short‐lived radiation products.^[^ ^78^ ^]^ However, such analysis is only feasible if long‐range effects are negligible, an assumption that itself requires validation. Moreover, while this approach enables assessment of whether beam effects influence an observation, it does not prove their absence.

We finally note that computational approaches can supply the reasoning for reaction mechanisms, not only for those dominated by radiation chemistry, but also for general materials studies, as elucidated elsewhere.^[^ ^211^, ^212^, ^213^ ^]^ Reaction throughput analysis is an example that works well with kinetic modeling to reveal the dominant reactions within a network.^[^ ^119^ ^]^

## 8 Outlook and Future Challenges

Significant progress has been made in revealing the effects of electron irradiation on LP‐TEM observations, but further questions remain to be solved. We highlight some of the key future challenges in the following paragraphs.

Regarding radiation chemistry, a key issue is the lack of direct experimental validation of *G*‐values under high dose rate electron irradiation. Recent reports suggest that the current set of values used, given in Table 1, may not fully represent the conditions seen in an LP‐TEM experiment. High yields of O_2_ were measured during ice radiolysis in cryo‐TEM at 93 K,^[^ ^138^ ^]^ which is not expected as a primary product (see Equation 4); electrochemical measurements confirmed H_2_ formation during liquid phase SEM but did not detect a notable amount of H_2_O_2,_ despite conflicting simulation results.^[^ ^169^ ^]^ EELS provides valuable insights into radiolysis product formation,^[^ ^91^, ^99^, ^138^ ^]^ and its relevance in radiolysis studies is expected to increase. However, quantitative EELS requires mitigation of energy loss by the membranes and multiple scattering. While this is straightforward in graphene LCs, ultrathin silicon nitride membranes^[^ ^214^, ^215^ ^]^ or regions with free‐standing graphene membranes in MEMS windows^[^ ^216^ ^]^ may be of synergetic use here. Control of the liquid layer thickness can be achieved in MEMS‐fabricated nanochannels,^[^ ^65^, ^136^ ^]^ or via pressure‐mediated control of window bulging in standard silicon‐based LCs.^[^ ^214^, ^217^, ^218^ ^]^ Thus, we speculate that with advanced hardware capabilities, fast in situ EELS experiments or correlative in situ photospectrometry might enable the measurement of primary products at the sub‐µs scale.

These disagreements between radiation chemistry simulations and measurements are contrasted by the good agreements of models with experimental observations discussed in this work. Hence, a model that bridges the gap between these findings is desired. Testing for the sensitivity of *G*‐value variations on the predictive power of such models would clarify the error margin induced by this uncertainty. In this context, STEM‐related effects should be distinguished from parallel beam illumination (TEM mode), mainly due to possible spur interactions or potentially not fully equilibrated primary species at short dwell times.

A further frontier for LP‐TEM modeling is the radiation chemistry of liquid systems beyond diluted aqueous systems. While the first promising approaches have been discussed within this work, current knowledge is far from comprehensive. The area is of key importance since battery research, biological systems, and indeed many liquid phase materials of interest rely on complex solutions. Understanding their behavior under irradiation is a prerequisite for quantitative investigations in these fields. We anticipate that further knowledge transfer from the radiation chemistry community will be highly beneficial.

We also expect that a more precise control of LP‐TEM conditions through liquid flow will be fruitful. As fast solution exchange systems evolve, we anticipate this will become a promising procedure to evaluate radiolysis effects and potentially tame artifacts during LP‐TEM, even beyond those that are beam‐induced.^[^ ^219^ ^]^ Control of liquid temperature is similarly important. Kinetic modeling facilitates accurate prediction of temperature effects on radiolysis, but the accuracy of *G*‐values and reaction kinetics in LP‐TEM should be extended to include their temperature‐dependent counterparts, particularly addressing cooling and possible quenching of radiolytic radicals.

Two other experimental artifacts require further work in the quest to improve the interpretation of LP‐TEM data. The first is beam‐induced heating, which so far is an under‐investigated topic. Experimental data is sparse and includes only spot checks of the parameter landscape of LP‐TEM. In addition, as the origin of beam‐induced heating is still under discussion, extrapolation of existing data should be performed carefully. Overconfident neglect is as dangerous as simply assuming that beam heating dominates shifts in the Gibbs free energy landscape. Instead, further systematic parameter studies spanning different systems would enhance the reliability of LP‐TEM significantly. The second experimental artifact we mention is hetero‐interface effects. SE emission, charging, heating, and chemical interactions are all possibilities that should be borne in mind when planning and conducting LP‐TEM experiments. The interplay of charging with radiation chemistry, in particular, requires greater investigation: future work could include electromigration in kinetic models or probe radiation chemistry interactions with a possible membrane charge‐induced Wien effect.

LP‐TEM has, since its origin, been seen as a unique probe of electrochemical processes.^[^ ^2^, ^19^, ^20^, ^22^, ^29^ ^]^ This leads to interesting questions regarding the interactions between radiation chemistry and electrochemical driving forces. Typical LC architectures allow direct observation of only a small fraction of the electrochemically active interface area, so in each experiment it is important to consider how representative are the dynamics observed under irradiation and how well they can be related to the electrochemically detected signal. It has been suggested that local irradiation can cause changes in the (globally averaged) electrochemical data,^[^ ^26^, ^169^, ^220^, ^221^ ^]^ as for instance is illustrated by the beam‐induced open circuit potential drop during liquid‐phase SEM displayed in Figure 13c. This demands further clarification. In this context, the effect of radiolysis should be assessed on electrochemical open circuit potentials, electrochemical double layers, electrolyte conductivity, and composition due to an increased concentration of ionic species and possible beam‐induced parasitic redox couples.

The reference electrode faces additional challenges when radiation chemistry must be considered: even reference electrodes close to the irradiated volume may not be sufficient to capture the actual in situ conditions. Improvements in reference electrode and electrochemical LC designs raise hope that these uncertainties will be overcome. Radiation chemistry may itself be useful in accelerated aging studies^[^ ^28^, ^222^, ^223^ ^]^ using the strategies discussed in this review.

We conclude by commenting that it should go without saying that any scientific finding must be reproducible, including simulations and data analyses. Providing all information to repeat these computations is mandatory alongside the raw data. This is often done most easily by enabling public access to the run file (which should be well‐documented) and all listed dependencies (such as software versions). In the case of custom‐made tools or scripts, the source code should consequently be made available to allow for reproducibility. Doing so helps not only to avoid scientific misconduct, but also accelerates research through the possibility of data recycling, which is particularly effective for resource‐intensive LP‐TEM studies.

## 9 Summary

In summary, LP‐TEM comes along with three main categories of irradiation‐induced artifacts: radiolysis, heating, and interactions with heterophases such as solid membranes, which can cause SE emission, charging, or additional interface reactions. Although these differ in importance depending on the specific experimental conditions, radiolysis is particularly ubiquitous during LP‐TEM. However, as discussed in this review, various handling strategies have been implemented that allow for quantitative conclusions regarding the magnitude of the beam‐induced effects. These strategies include (kinetic) modeling, mitigation techniques, and reference experiments, ideally applied in combination. When performed appropriately, these strategies enhance LP‐TEM, transforming it into an even more powerful analysis tool with the potential to revolutionize nanoscience in liquid media.

## Conflict of Interest

The authors declare no conflict of interest.

## Data Availability

The data that support the findings of this study are available from the corresponding author upon reasonable request.
